# Protein degradation and dynamic tRNA thiolation fine-tune translation at elevated temperatures

**DOI:** 10.1093/nar/gkv322

**Published:** 2015-04-13

**Authors:** Kshitiz Tyagi, Patrick G.A. Pedrioli

**Affiliations:** 1 MRC Protein Phosphorylation and Ubiquitylation Unit, College of Life Sciences, University of Dundee, Dundee DD1 5EH, UK; 2 The Swiss Federal Institute of Technology, The Institute of Molecular Systems Biology, Auguste-Piccard-Hof 1, 8092 Zurich, Switzerland

## Abstract

Maintenance of protein quality control has implications in various processes such as neurodegeneration and ageing. To investigate how environmental insults affect this process, we analysed the proteome of yeast continuously exposed to mild heat stress. In agreement with previous transcriptomics studies, amongst the most marked changes, we found up-regulation of cytoprotective factors; a shift from oxidative phosphorylation to fermentation; and down-regulation of translation. Importantly, we also identified a novel, post-translationally controlled, component of the heat shock response. The abundance of Ncs2p and Ncs6p, two members of the URM1 pathway responsible for the thiolation of wobble uridines in cytoplasmic tRNAs tK^UUU^, tQ^UUG^ and tE^UUC^, is down-regulated in a proteasomal dependent fashion. Using random forests we show that this results in differential translation of transcripts with a biased content for the corresponding codons. We propose that the role of this pathway in promoting catabolic and inhibiting anabolic processes, affords cells with additional time and resources needed to attain proper protein folding under periods of stress.

## INTRODUCTION

Robust and rapid response to ever changing extracellular environmental conditions is a prerequisite for the survival of all life forms. In *Saccharomyces cerevisiae* the environmental stress response (ESR) comprises approximately 900 genes (1,2). The mechanism, timing and intensity of ESR gene expression is tied to the kind and severity of the stress (3). Complementing this common expression program, several genes are distinctly controlled by specific stress conditions. In addition to the transcriptional regulation, post-translational modifications also play key roles in sensing the stress, transducing signals and affecting the ESR gene expression program (4–6). Under stress conditions cells also regulate the abundance of ribosomes and tRNAs to slow down translation (1–2,7). Additionally, proteins such as the translation initiation factors and ribosomal structural proteins are post-translationally modified to modulate their activity (8–10).

RNA, the main constituent of the translational apparatus of a cell, carries a wide array of post-transcriptionally modified nucleotides (11–13). These modifications increase the repertoire of otherwise limited inter and intra molecular interactions that the four canonical nucleotides can make (14). Among all RNA types, tRNAs exhibit the maximum diversity of modified nucleotides with >90 documented variants (15,16). Even though the structures and biosynthesis for the majority of these nucleotide modifications have been characterized (14,17), their role and regulation is still poorly understood. Recently, it was reported that in *S. cerevisiae* exposure to chemicals causing oxidative and/or DNA-damage stress changes the levels of several tRNA modifications (18). Overall, it is likely that several of these nucleotide modifications are important for the functioning of RNAs and to regulate their activity.

The ESR, together with the ubiquitin proteasome system, is key in maintaining proteostasis. With the increase in average life span, diseases associated with reduced protein homeostasis are increasingly burdening the health of our society. It is estimated that Alzheimer's and Parkinson's diseases alone, two age-of-onset neurodegenerative diseases associated with the formation of toxic protein aggregates, affect tens of millions of people world-wide (19). Understanding the mechanisms controlling formation and prevention of these aggregates is quickly becoming one of the great medical challenges of our century. Heat stress signalling pathways that are triggered in response to heat shock, collectively known as the heat shock response (HSR), are amongst the best studied component of the ESR (20,21). The transcriptional response to heat stress, has been extensively studied at the system level using micro-arrays (1,2), and was found to heavily rely on the action of the transcription factors Hsf1p and Msn2p/Msn4p. Here, we present the first comprehensive analysis of the differential proteome composition of yeast cells exposed to mild, long-term heat stress.

## MATERIALS AND METHODS

Further details on protocols are described in Supplementary Materials and Methods.

### Yeast strains

Yeast strains used in this study are described in Supplementary Table S1.

### SILAC labelling and mass spectrometry analysis

Yeast cells auxotrophic for Lys and Arg were grown and labelled with SILAC using ^13^C_6_,^15^N_4_-Arg and ^13^C_6_,^15^N_2_-Lys, lysed and their proteins extracted as previously described (22). Digestion for MS analysis was done using the FASP procedure (23) with sequencing grade modified trypsin (V5113, Promega). Digested peptides were C18 purified and analysed by LC–MS–MS/MS using an UltiMate 3000 UHPLC system (Dionex, Thermo Scientific) directly coupled to an LTQ-Orbitrap Velos Pro mass spectrometer (Thermo Finnigan). After the acquisition, MS data were processed and analysed by the trans-proteomic pipeline to identify proteins and estimate their abundance ratios as described in (22), except Comet was used as the peptide database search engine (24).

### Quantitative PCR

Quantitative real time PCR analyses were performed with the predesigned TaqMan gene expression assays on Applied Biosystems 7900HT Fast Real-Time PCR System.

### Western blotting

Antibodies used for the western blot analyses were mouse-anti-HA antibody (HA.11 Clone 16B12; MMS-101R, Covance), mouse-anti-actin antibody (ab8224, Abcam) and goat-anti-mouse IgG, IgA, IgM (H + L) horseradish peroxidase conjugate (A-10668, Invitrogen).

### APM-dPAGE and northern blot

The procedure used was essentially as described previously (25). Bulk tRNA were electrophoresed through a 10% polyacrylamide gel with 7 M urea, 0.5× TBE and 50 μg/ml [(*N*-acryloylamino)phenyl] mercuric chloride (APM) and either imaged directly with 1:10 000 SYBR gold (Invitrogen) or transferred to a nylon membrane and probed with oligonucleotide probes labelled with γ-^32^P ATP.

### Purification of specific tRNAs and RNA MS

Individual tRNAs were purified using the procedure described in (25), digested and dephosphorylated to individual nucleosides and analysed by LC–MS–MS/MS.

The mass spectrometry data have been deposited to the ProteomeXchange Consortium (http://proteomecentral.proteomexchange.org) via the PRIDE partner repository with the dataset identifier PXD001864.

## RESULTS

### Differential proteome composition of yeast grown at 30°C versus 37°C

**Figure 1.**
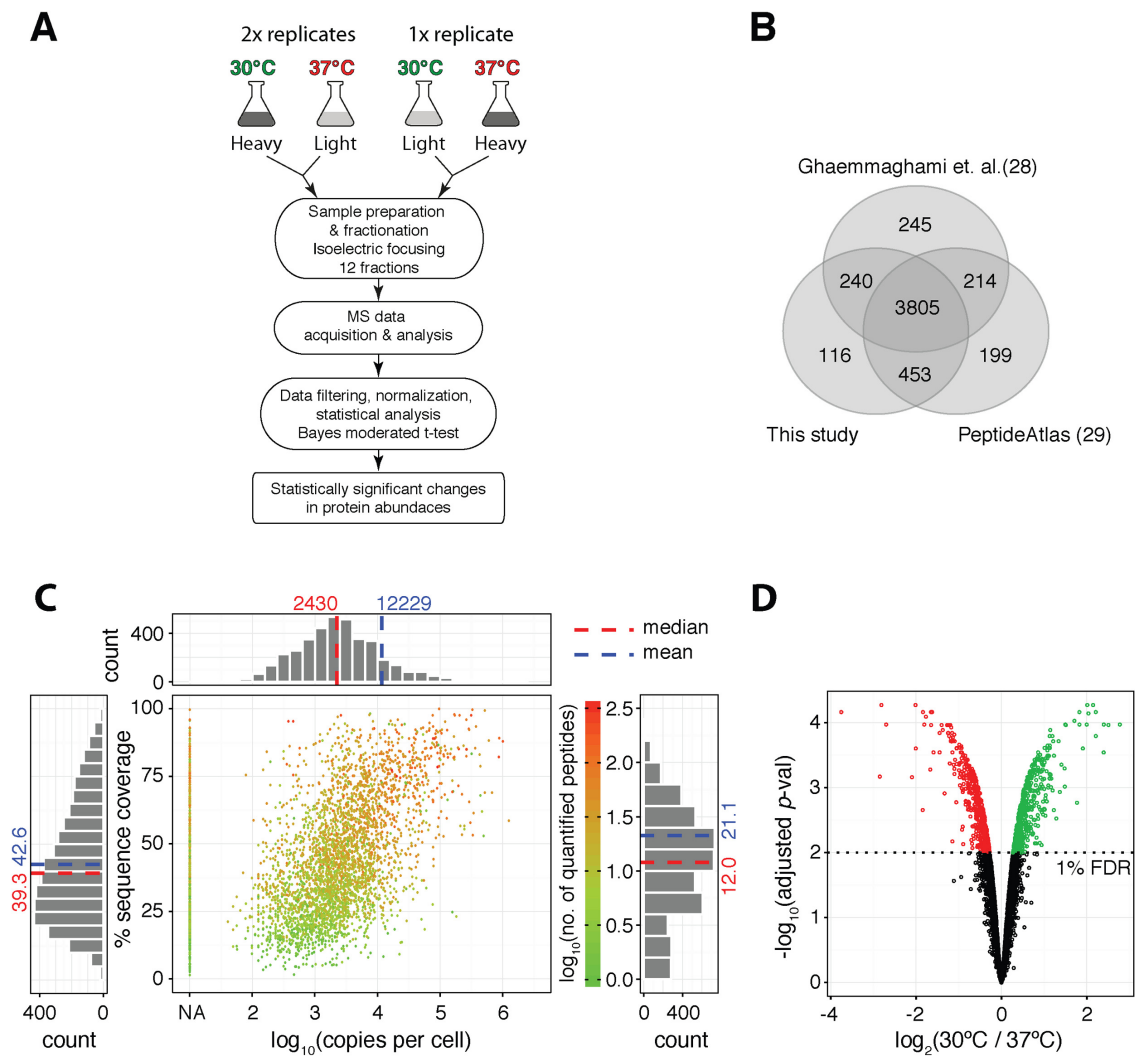
Quantitative proteomics of the heat stress response. (**A**) Schematic representation of the workflow. (**B**) Overlap of the proteins quantified in this and two other large scale yeast studies (28,29). (**C**) Graphical summary of sequence coverage and dynamic range achieved in the experiment. Copy per cell values are from (28). (**D**) Volcano plot representation of the significance of protein fold changes. Dotted line: 1% FDR; red and green circles highlight the significantly up- and down-regulated proteins, respectively.

To study the differential proteome composition of yeast cells grown at 30°C versus 37°C, we used SILAC-based quantitative proteomics. Three biological replicates were grown and analysed using a label switch workflow that alternated the growth condition labelled with SILAC heavy residues. Each sample was fractionated into 12 fractions using isoelectric focussing with OffGel electrophoresis to help increase proteome coverage (Figure 1A).

This led to the identification of 4663 proteins at a ProteinProphet (26) calculated 1% false discovery rate (FDR). A total of 4612 of these were also quantified based on at least one peptide with probability higher than the PeptideProphet (27) calculated minimum probability threshold of 1% FDR. This represents 68% of the predicted yeast proteome Table 1. Interestingly, even by combining multiple large scale datasets (28,29) (Figure 1B) only 78.4% of the predicted proteome can be accounted for. It is possible that the remaining proteins are only expressed under specific growth conditions, or that their abundance is below detection levels. We however note that our analysis spanned five orders of magnitude in protein abundances (Figure 1C). Protein coverage positively correlated to log_10_ of the protein copies per cell values (*r* = 0.56) and had a mean value of 42.64% across the whole dataset. On average each protein was quantified by 21 high confidence peptides.

**Table 1.** Proteomics performance

	SGD	MS based this study (% of SGD)	Tag-based Ghaemmaghami *et al*. (28)	MS-based PepAtlas (29)
Total	6717	4612 (68.66)	4504	4668
Verified	4939	4198 (85)	4048	4197
Uncharacterized	853	401 (47.01)	409	402
Dubious	810	4 (0.49)	41	11
Transposable element	89	4 (4.49)	3	55
Pseudogene	26	5 (19.23)	3	3

Comparison of the number of proteins quantified in the present study against the number of proteins from: the Saccharomyces Genome Database (SGD); the tag based proteomics by Ghaemmaghami *et al*. (28); and the Peptide Atlas repository (29). Note that the number of proteins reported for the tag based studies is 13 less than originally indicated in Ghaemmaghami *et al*. (28), since those entries were either deleted from SGD or merged into existing ORFs.

False positive peptide assignments, noise, and overlapping signals in one of the isotopic channels, can lead to erroneous protein quantification. These type of errors are often addressed by switching the isotopic labels across the samples (30,31). Here, we devised a workflow that takes advantage of the label switch information at the peptide, rather than at the protein level. More specifically, only proteins with at least two high confidence peptides quantified across label switched samples were retained. Furthermore, only peptides consistently quantified in across labelling designs (i.e. $\left| {{\rm log}_2 \left( {R_1 } \right) - {\rm log}_2 \left( {R_{ - 1} } \right)} \right| {<} \sqrt 2 \times 0.4$) were used for the calculation of relative protein abundances (Supplementary Figure S1A and B). The resulting dataset was devoid of systematic biases both at the peptide and protein levels (Supplementary Figure S1C–E). The stringently filtered dataset was comprised of 3614 proteins (Supplementary Table S2) and retained good coverage of the dynamic range of protein expression (Supplementary Figure S1F).

Statistically significant changes in protein abundances were calculated by empirical Bayes moderated *t*-test (32,33) and are shown in Figure 1D. Out of 1007 significantly changing proteins, 488 were more and 519 were less abundant at the elevated temperature. 83.5% of the 115 significantly changing proteins in common with the heat shock study by Nagaraj *et al*. (34) were positively correlated (Supplementary Figure S2A). Next, we selected three micro-arrays with matching experimental conditions from the transcriptomics analysis of yeast cells in steady state growth at elevated temperature by Gasch *et al*. (2) and calculated significant mRNA abundance changes (1% FDR) using a Bayes moderated *t*-test. Overall, 89.5% of the significant changes at the proteome and transcriptome levels were positively correlated (Supplementary Figure S2B).

**Figure 2.**
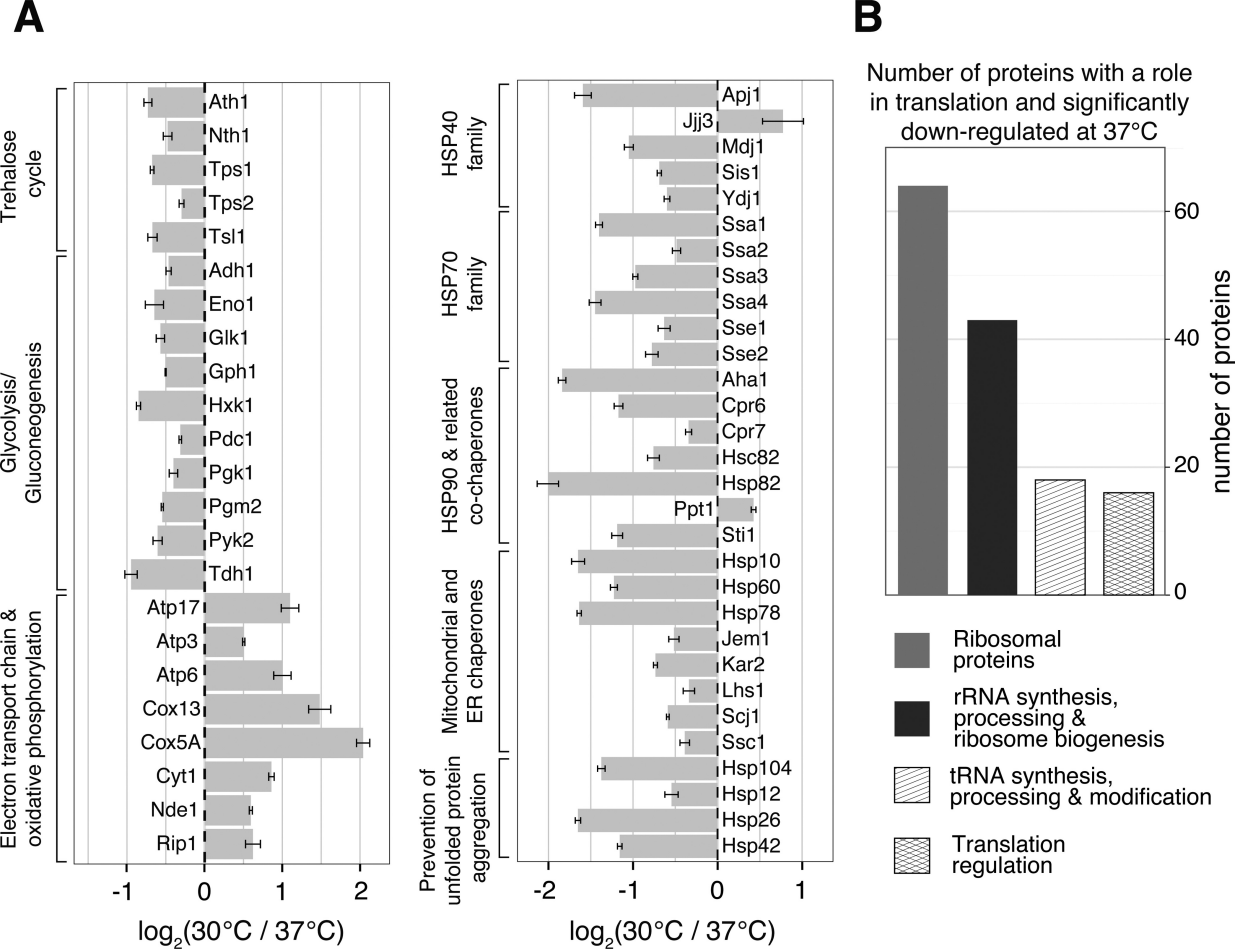
Selected candidates proteins with altered abundance at 30°C versus 37°C. (**A**) Protein abundance changes for some of the proteins with important roles in the response to heat stress. Error bars represent standard error. (**B**) Number of proteins with a role in translation and significantly down-regulated at 37°C.

We also manually validated key-proteins in the response to elevated temperatures Figure 2A. The abundance of many heat shock proteins (HSPs) increases in the presence of proteotoxic stress. The SSA subfamily of HSP70s (i.e. Ssa1p, Ssa2p, Ssa3p and Ssa4p), responsible for binding hydrophobic regions of unfolded proteins and promoting their folding, was significantly up-regulated. HSP70s use ATP to promote folding and rely on the HSP110s (Sse1p and Sse2p) nucleotide exchange family. Although, it has previously been reported that Sse2p abundance increases significantly more than that of Sse1p (2,35), under the mild stress conditions we used, the relative increase in the abundance of these proteins was similar. HSP40s increase HSP70s ATP hydrolysis rate. Ydj1p, Sis1p, Mdj1p and Apj1p were up-regulated at 37°C, in contrast, Jjj3p was down-regulated. These observations are consistent with transcriptomics findings (2) and support the idea that Jjj3p, with its role in diphtamide synthesis, might play a specialized role as an HSP40 (36). The abundance of HSP90s (Hsc82p and Hsp82p), which help in protein maturation, was increased at elevated temperatures. HSP90s co-chaperones Sti1p, Aha1p, Cpr6p and Cpr7p were also up-regulated, whereas Ppt1p was down-regulated. Although, 37°C does not cause protein aggregation (37), we found that Hsp104p a disagregase that helps resolubilize protein aggregates, as well as Hsp26p, Hsp42p and Hsp12p, responsible for keeping denatured proteins in solution, were significantly up-regulated. Finally, several mitochondrial and endoplasmic reticulum chaperones were also up-regulated (e.g. Ssc1p, Hsp78p, Hsp60p, Hsp10p, Kar2p, Lhs1p, Scj1p, Jem1p).

Trehalose stabilizes proteins against heat-induced denaturation (38–40). Tps1p, Tps2p and Tsl1p, which together with Tps3p form the yeast trehalose synthase were all significantly up-regulated at 37°C. Furthermore, enzymes responsible for the synthesis of the trehalose precursors glucose-6-phosphate (G6P) and uridine–diphosphate–glucose (UDPG) were also up-regulated (e.g. Hxk1p, Glk1p, Pgm2p and Gph1p). Interestingly, we also found up-regulation of the trehalases Nth1p and Ath1p. Overall, the changes in trehalose cycle proteins were in good agreement with previously reported changes in gene expression levels (1,2).

Taking a broader view on carbon metabolism, we observed up-regulation of glycolysis and fermentation pathways (e.g. Tdh1p, Pgk1p, Gph1p, Eno1p, Pyk2p, Pdc1p, Adh1p), and down-regulation of oxidative phosphorylation (e.g. Cox5Ap, Cox2p, Cox4p, Qcr7-8p, Qcr10p, Cox13p, Atp1-7p and Atp17-20p).

A significant part of the ESR focuses on down-regulating genes involved in protein synthesis. Based on their Saccharomyces Genome Database (SGD) description, more than 140 out of the 519 significantly down-regulated proteins in our dataset are involved in translation. We observed a significant enrichment of cytoplasmic and mitochondrial ribosomal proteins (e.g. Rpl28-30p, Rpl6A-9Ap, Rps23A-Bp, Rps24A-Bp, Mrps16-18p, Mrpl6-8p), proteins participating in ribosome biogenesis (e.g. Nog1-2p, Nop13-16p), tRNA synthetases (e.g. Ala1p, Ths1p, Ils1p, Cdc60p, Deb81p) and translation regulation (Figure 2B).

Overall, FunSpec (41) analysis of the over-represented GO terms and MIPS functional classes (Supplementary Figure S2C-D and Supplementary Tables S3 and S4) highlighted down-regulation of translation and energy production (e.g. mitochondrial translation; translation; ribosome biogenesis; ATP biosynthetic process) and an up-regulation of stress associated processes (e.g. protein folding; oxidation-reduction process; response to stress).

**Figure 3.**
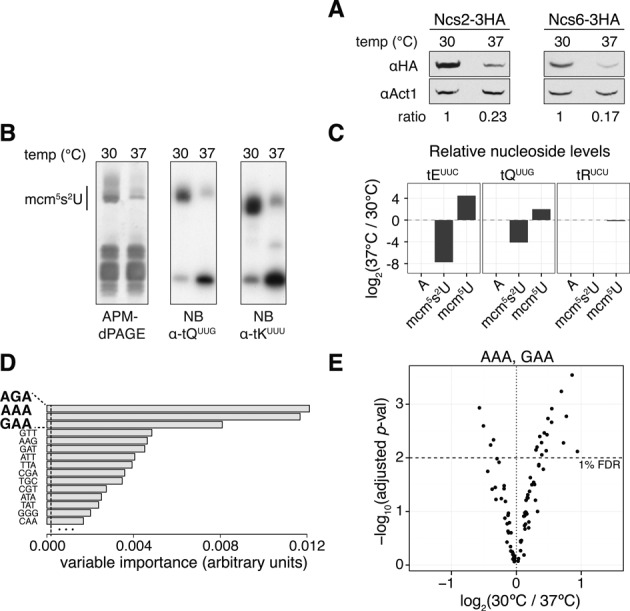
URM1 pathway activity is down-regulated at elevated temperatures. (**A**) Western blot validation of Ncs2p and Ncs6p down-regulation. (**B**) APM-dPAGE and northern blot analysis of the bulk tRNA isolated from the cells grown under indicated conditions. (**C**) Relative changes in abundance of nucleosides as measured by RNA-MS analysis. tRNAs were either purified from cells grown at 37°C or 30°C. Adenosine is shown as a control. (**D**) Random forest calculated importance of codon abundance, in separating proteins in up- and down-regulated sets. Vertical dashed line: absolute value of the lowest predictor. (**E**) Protein fold changes versus significance values for genes with the 2% highest frequency of the indicated codon. Horizontal dashed line (*y* = 2): 1% FDR.

Amongst the significantly changing proteins not previously associated with the HSR, we noticed members of the URM1 pathway (see Table 2 and Supplementary Figures S3–S5). This pathway, comprised of Tum1p, Uba4p, Urm1p, Ncs2p and Ncs6p, is responsible for generating 2-thiouridine (s^2^U_34_) at the wobble position of the three eukaryotic cytoplasmic tRNAs tK^UUU^, tQ^UUG^ and tE^UUC^. At the higher temperature Uba4p was up-regulated by 1.49-fold, whereas Ncs2p and Ncs6p were down-regulated by 1.61- and 4.03-fold, respectively (Figure 3A for western blot validation). This suggested that URM1 pathway-modified tRNAs might be hypothiolated at 37°C. Interestingly, similarly to the HSR, loss of URM1 pathway activity leads to down-regulation of anabolic and up-regulation of catabolic processes (22) (Supplementary Figure S2C and D).

**Table 2.** URM1 pathway under heat stress

Protein name	log_2_(30°C/37°C)	Adj. *p*-value
Tum1p	−0.03	0.7328
Uba4p	−0.58	0.0012
Ncs2p	0.69	0.0020
Ncs6p	2.01	0.0001
Urm1p	NA	NA

Protein abundance ratios for the URM1 pathway members in cells grown at 30°C and 37°C. Urm1p was not present in the stringently filtered dataset as it was quantified by only one high confidence peptide. Manual validation of this peptide quantification (Supplementary Figure S3) confirmed that it was not significantly changing across the three biological replicates.

### tRNA wobble uridine residues in tK^UUU^, tE^UUC^ and tQ^UUG^ are differentially modified in yeast grown at elevated temperatures

To test for this hypothesis we compared tRNA thiolation levels at 30°C versus 37°C using APM ([*p*-(*N*-acrylamino)-phenyl]mercuric chloride) supplemented denaturing PAGE (APM-dPAGE). APM retards the migration of thiolated tRNAs, which appear as higher molecular weight species in the gel. As shown in Figure 3B we found a remarkable reduction of thiolation at elevated temperature. Interestingly, the reduction in thiolation was proportional to the duration of incubation at the elevated temperature (Supplementary Figure S6A).

tRNA wobble s^2^U_34_ residues are further modified by the elongator (ELP) pathway to form 5-methoxycarbonylmethyl-2-thiouridine (mcm^5^s^2^U_34_). Interestingly, a cross-talk exists between the two pathways (Supplementary Figure S6B) (25). To assess whether hypothiolation at 37°C was caused by a change in ELP pathway activity we digested and dephosphorylated individual tRNA species (Supplementary Figure S6C) and analysed them by RNA-MS (Supplementary Figure S7). Whereas wobble uridines in tE^UUC^ and tQ^UUG^ are doubly modified to mcm^5^s^2^U_34_, those in tR^UCU^ are singly modified to mcm^5^U_34_. As shown in Figure 3C and Supplementary Figures S8–S10, mcm^5^s^2^U levels were greatly reduced in the tRNA species modified by the URM1 pathway. Furthermore, the ELP pathway activity was unperturbed at 37°C as indicated by the unchanged modification levels of tR^UCU^. Therefore, the increase in mcm^5^U in tE^UUC^ and tQ^UUG^ resulted from the accumulation of a precursor that is normally further modified to mcm^5^s^2^U_34_. A similar effect has been reported in *urm1Δ* mutants (25,42).

In the absence of tRNA thiolation a subset of the proteome highly biased in the content of AAA, CAA, AAG and GAA codons is less efficiently translated (22). Here, we separated significantly changing proteins in the 30°C versus 37°C dataset in up- and down-regulated ones and used a random forest classifier to identify the codons that best predicted membership to one of the two classes. As shown in Figure 3D, AAA and GAA, whose corresponding tRNAs are thiolated in a URM1 pathway dependent manner, were amongst the top three most important codons. Furthermore, proteins whose transcripts were biased in the content of these codons were down-regulated at 37°C (see Figure 3E and Supplementary Figure S11). AGA was also amongst the top codons. In yeast, the corresponding tRNA is modified by the ELP, but not by the URM1 pathway. However, as we have shown, the activity of the ELP pathway was unaffected at elevated temperatures. Interestingly, in higher eukaryotes, such as bovines (43), mice (44), and humans (see Supplementary Figure S12), tR^UCU^ wobble uridines are thiolated.

**Figure 4.**
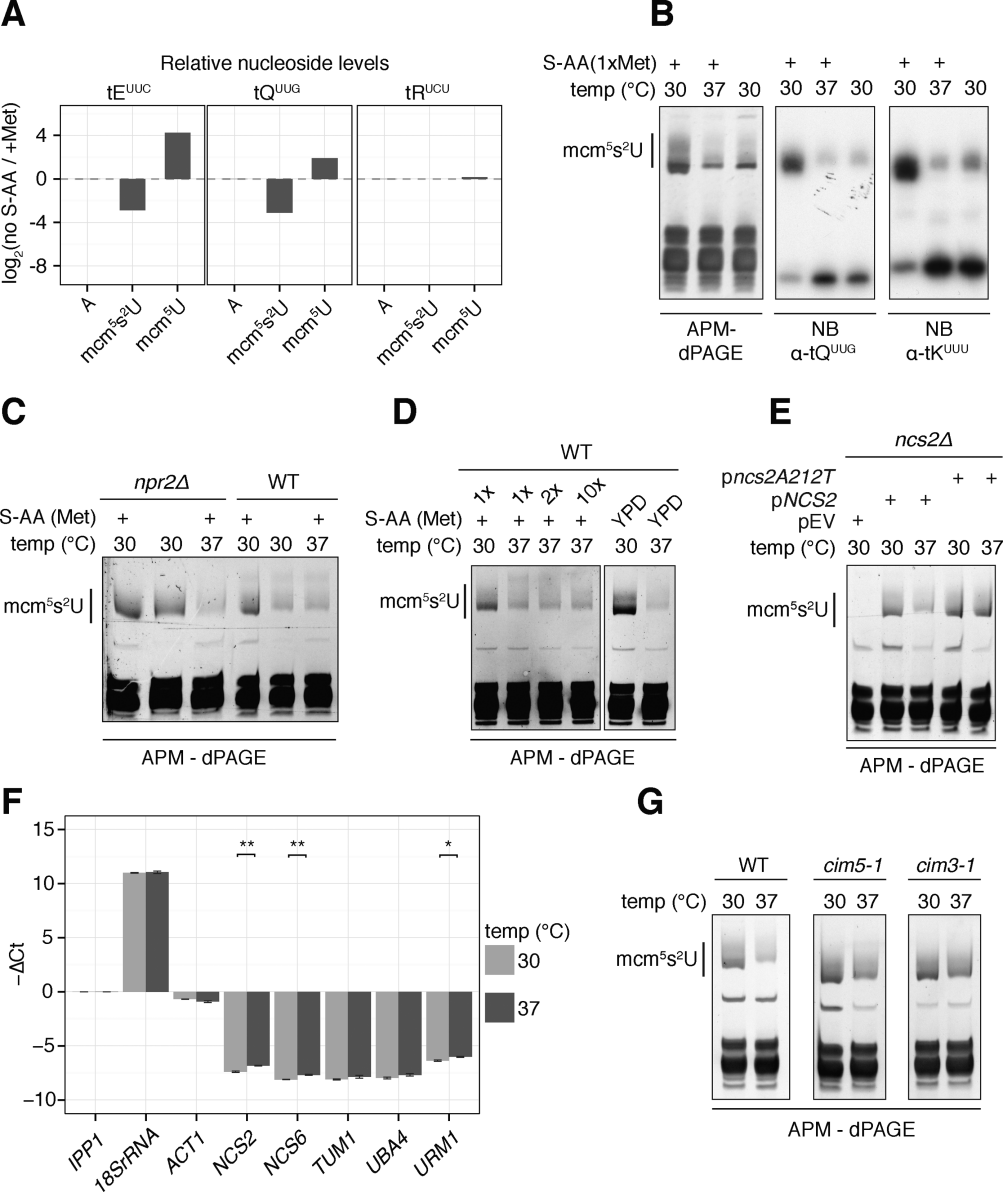
Independent mechanisms regulate tRNA thiolation upon S-AA starvation and growth at elevated temperature. (**A**) Relative changes in abundance of nucleosides as measured by RNA-MS analysis. tRNAs were either purified from cells starved for S-AA (no S-AA) or in the presence of methionine (+Met). Adenosine is shown as a control. (**B**) APM-dPAGE or northern blot analyses indicate a similar reduction in thiolation in tRNA isolated from cells starved for S-AA or grown at elevated temperature. (**C**) *npr2Δ* prevents hypothiolation in cells starved for S-AA, but not in cells grown at 37°C. (**D**) Hypothiolation at 37°C does not depend on S-AA content. (**E**) *ncs2_A212T* prevents hypothiolation at 37°C, but not in S-AA starved cells. (**F**) Mean ΔCt values for three biological replicates of quantitative real time PCR measurements for URM1-pathway mRNA levels in cells grown at 37°C or 30°C. The mean ΔCt value of *IPP1* was used for normalization. 18S rRNA and *ACT1* were analysed as negative controls. (*) and (**) indicate *P*-values of less than 0.05 and 0.01, respectively, obtained by a two-sample *t*-test of the difference between the ΔCt values at 30°C versus 37°C for a given gene. Error bars represent standard error (*n* = 3). (**G**) APM-dPAGE analysis for tRNA extracted from wild-type, *cim5-1* and *cim3-1* yeast cells grown at either 30°C or 37°C.

Laxman *et al*. (45) have recently shown that tRNA hypothiolation in cells starved for sulfur containing amino acids (S-AA) can be rescued by deleting *NPR2*. To test if hypothiolation in cells grown at elevated temperatures is controlled in a similar fashion, we first analysed the down-regulation of thiolation in S-AA starved cells by RNA-MS and northern blot analysis (Figure 4A and B). This indicated that S-AA starvation and growth at elevated temperatures had similar effects on tRNA modification. However, although S-AA starvation induced tRNA hypothiolation was effectively suppressed in an *npr2Δ* strain (Figure 4C compare change in thiolation level between lanes 1, 2 (*npr2Δ* background) to that between lanes 4, 5 (wt background)), the same was not the case for temperature induced hypothiolation (Figure 4C compare change in thiolation level between lanes 1, 3 (*npr2Δ* background) to that between lanes 4, 6 (wt background)). Furthermore, the latter was not rescued by methionine supplementation, nor by growing the cells in a complex rich media (Figure 4D).

A single nucleotide polymorphism in *NCS2* (i.e. A212T; H71L) causes a high temperature growth phenotype (46). We tested if this polymorphism was associated with the regulation of tRNA thiolation at 37°C. We transformed an *ncs2Δ* background with either the wild-type copy of *NCS2* or the *ncs2A212T* point mutant and assessed tRNA thiolation by APM-dPAGE. As shown in Figure 4E, whereas thiolation levels in strains expressing either the wild-type or H71L variant of Ncs2p were similar at 30°C (lanes 2 and 4), at 37°C the H71L allele did not exhibit reduced tRNA thiolation (lanes 2, 4 and 5). On the other hand, *ncs2A212T* was unable to rescue the decrease in thiolation levels caused by sulfur amino acid starvation (Supplementary Figure S13).

As expected, quantitative real-time PCR, revealed that none of the URM1 pathway components were down-regulated at the transcriptional level (Figure 4F). We therefore used *cim5-1* and *cim3-1*, two temperature sensitive mutants of proteasomal ATPases, to probe for a role of proteasomal activity in the differential thiolation. These mutants have proteasomes that are fully active at 30°C, but only partially active at 37°C (47). As shown in Figure 4G, these strains exhibited a much less severe reduction in thiolation when switched to the non-permissive temperature. Importantly, this indicates that the reduction in activity is not caused by unspecific heat-inactivation of URM1 pathway components.

Overall, we found that in yeast exposed to prolonged heat stress the activity of the URM1 pathway, but not that of the ELP pathway, was reduced at the post-translational level in a proteasomal dependent fashion. This, in turn resulted in hypothiolation of tK^UUU^, tE^UUC^, tQ^UUG^ and reduced abundance of proteins whose transcripts are highly biased in AAA and GAA codons. Intriguingly, this response relied on a different mechanism and impinged on different components (i.e. Ncs2p, and Ncs6p versus Uba4p and Urm1p) of the URM1 pathway than S-AA starvation induced hypothiolation.

### tRNA over-expression can compensate for reduced thiolation levels

**Figure 5.**
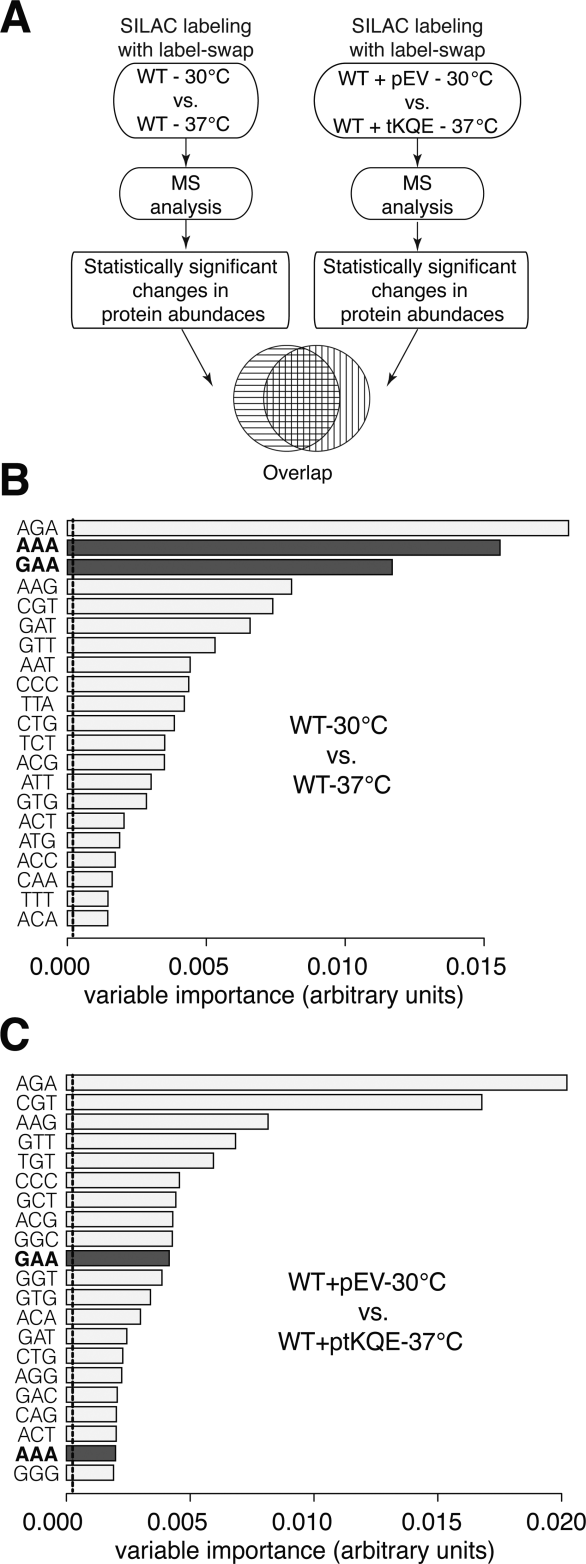
tRNA over-expression compensates for reduced thiolation at 37°C. (**A**) Schematic representation of the proteomics workflow. (B and C) The relative importance of each codon in predicting up- or down-regulation of a protein was calculated using a random forest algorithm. The relative importance of codon abundance is shown for (**B**) the 30°C versus 37°C dataset and (**C**) the tRNA over-expression dataset. Longer bars are associated with codons that are better at predicting changes in the corresponding proteins.

It has been previously shown that over-expression of the unmodified tRNAs tK^UUU^, tE^UUC^, tQ^UUG^ can rescue phenotypes associated with URM1 and the ELP pathway mutants (25,48). Additionally, we have shown that this is sufficient to rescue protein expression levels in a tRNA thiolation deficient strain (22). Since the over-expression of the three tRNAs did not affect their thiolation levels (Supplementary Figure S14), we took advantage of this to deconvolute the impact of differential tRNA modification and other mechanisms on controlling proteome composition during growth at elevated temperatures. Specifically, we compared the relative abundance of proteins in wild-type yeast grown at 30°C to that in wild-type yeast over-expressing the three thiolated tRNAs (ptKQE) grown at 37°C (Figure 5A).

Three biological replicates were analysed similarly to what was done for the first proteomics analysis. The resulting data were normally distributed and devoid of biases both at the peptide and protein levels (Supplementary Figure S15). Overall, 1957 proteins were quantified.

To minimize possible artefacts arising from different proteome subsets having been observed, only the 1881 proteins identified in both the 30°C versus 37°C and in the 30°C versus 37°C + ptKQE datasets were further analysed. A total of 547 proteins (274 up and 273 down) in the normal dataset and 611 (322 up and 289 down) in the ptKQE dataset were found to be of significantly different abundance (Supplementary Table S5). Overall, protein abundances in both datasets were highly correlated (r = 0.86). However, a random forest analysis on the significantly up- and down-regulated protein sets highlighted important differences (Figure 5B and C). Even though AAA and GAA codons still played a major role in discriminating down-regulated proteins in the smaller 30°C versus 37°C subset (see panel B), over-expression of tRNAs tK^UUU^, tE^UUC^, tQ^UUG^ reduced the impact of these codons in controlling proteome composition at elevated temperature (see panel C).

To characterize the subset of the proteome affected by tRNA hypothiolation, we looked at genes with the 10% highest frequency in the genome for at least one of the AAA, CAA and GAA codons and whose corresponding proteins were significantly (1% FDR) down-regulated in the 30°C vesus 37°C but not in the 30°C versus 37°C + ptKQE datasets (Supplementary Table S6). Interestingly, this list is rich in ribosomal proteins, and proteins involved in ribosomal biogenesis. Components of oxidative phosphorylation pathway, such as the α and β subunits of the F0F1-ATP synthase are also present.

Overall, these analyses indicated a causality relationship between tRNA hypothiolation at elevated temperatures and the reduced translation of mRNAs highly biased in the content of AAA and GAA codons. The proteome subset rescued by tRNA over-expression was consistent with our initial observation that translation and oxidative phosphorylation are reduced in yeast grown at elevated temperature and showed that down-regulation of these processes is controlled, at least in part, via differential translation.

## DISCUSSION

We investigated changes in the proteome of yeast grown at elevated temperatures. The expression levels of hundreds of genes are affected by environmental stresses. Often these changes are proportional to the stress, peak early and return to near-normal once the cells have adapted (1,2). Here we found that, even after extended exposure, HSPs levels remained elevated at 37°C compared to 30°C. Additionally, although we choose a mild heat challenge (21,37), HSPs responsible for resolubilizing protein aggregates such as Hsp104p were up-regulated. Interestingly, Hsp104p has been implicated in acquired heat tolerance (49,50). Our data suggest that this protein might be pre-emptively up-regulated to protect against a further temperature increase. More generally, it has been suggested that the HSR evolved to prevent future damage, rather than to recover from an existing one (21,51). For instance, a mild heat shock confers protection against oxidative stress damage (21 and references therein, 52). We found that at 37°C enzymes in the glycolytic and fermentation pathways were up-regulated whereas components of the electron transport chain and F0F1-ATP synthase were down-regulated. Down-regulation of oxidative phosphorylation, reduces an important intracellular source of free radicals, and might contribute to the acquired resistance to oxidative stress (53). Furthermore, this allows for fast, albeit less efficient, production of ATP, and might help in quickly meeting the energy requirements of HSPs. Importantly however, *S. cerevisiae* is a Crabtree positive organism and metabolomic studies have found that only a marginal amount of oxidative phosphorylation takes place in the presence of glucose (54). Hence, cellular respiration components might be down-regulated in preparation of possible changes in the available carbon source. The trehalose cycle offers further support to the idea that cells growing at elevated temperatures are primed to respond to future changes in growth conditions. Trehalose is key in the response to heat, but can interfere with protein folding at elevated concentrations. Interestingly, enzymes responsible for trehalose synthesis have a higher temperature optimum than those responsible for its degradation (55,56). Our data indicate that both sets of enzymes are up-regulated at 37°C. This setup ensures that a change in temperature, in either direction, is sufficient to quickly shift the balance of the cycle.

Although transcriptomics studies have afforded us a deep insight into the HSR, post-translational responses can not be inferred from these studies. By measuring differential proteome composition in cells growing at 30°C versus 37°C, we identified a novel, post-translationally regulated aspect of the HSR. Specifically, the activity of the URM1 pathway, which is responsible for thiolating the wobble uridines of the three eukaryotic cytoplasmic tRNAs tK^UUU^, tQ^UUG^ and tE^UUC^, is down-regulated in yeast grown at 37°C. s^2^U_34_ is important for efficient ribosomal A-site binding and translation of mRNAs biased for AAA, CAA, GAA and AAG codon content (22). As we were revising this manuscript, others (57,58) have also reported, using complementary approaches, a temperature-dependent reduction in tRNA thiolation. Using tagged proteins, Damon *et al*. (57) found that the abundance of all members of the URM1 pathway (with the possible exception of Urm1p, which was not tested) and some members of the elongator pathway was affected. Here we found that, similarly to what was found by Han *et al*. (58), when using endogenous proteins the activity of the elongator pathway remained unchanged and only the abundances of Ncs2p and Ncs6p were significantly down-regulated at 37°C. While previous studies of the URM1 pathway relied on artificially inactivating the activity of the pathway, this is the first study in which the differential proteome composition has been monitored under a physiological condition that results in reduced tRNA thiolation. Interestingly, under this growth conditions we find that AAA and GAA codons play a more important role than CAA ones, which in turn might reflect differences in the composition of the transcriptome. The translational machinery in particular (Supplementary Table S7) is highly biased in the content of codons recognized by s^2^U_34_ containing tRNAs and is down-regulated in a thiolation deficient mutant (22,45). Consistently, here we find that in the HSR the URM1 pathway contributes to the down-regulation of ribosomal proteins and of proteins involved in ribosomal biogenesis. Ribosomal genes are also rich in AGA codons. Although the corresponding tRNA is not modified by the URM1 pathway in yeast, it is therefore not surprising that the codon was identified in our unbiased analysis of codon significance. Interestingly, higher eukaryotes might have evolved thiolation of tR^UCU^ to increase the control that tRNA thiolation has on modulating the translational capacity of a cell. The URM1 pathway has recently been shown to play a role in the response to sulfur amino acid starvation (45), suggesting that it might have a more general role in the ESR. Intriguingly, we find that the activity of the pathway is distinctively controlled under these two conditions. First, in the HSR Ncs2p and Ncs6p are down-regulated, whereas in S-AA starvation Uba4p and Urm1p are. Not surprisingly, an NCS2 SNP, which confers a growth advantage at elevated temperatures, rescues hypothiolation induced by elevated temperature, but not by S-AA starvation. Second, while tRNA thiolation in an *npr2Δ* mutant is no longer sensitive to S-AA starvation, *npr2Δ* yeast grown at elevated temperatures remains sensitive. Over-expression of unmodified tRNAs can rescue the differential proteome composition of an *urm1Δ* strain (22). Similarly, here we found that tRNA over-expression decreases the importance of URM1 pathway codons at elevated temperatures. However, consistently with changes in gene expression programs playing a major role in the HSR, the proteome of yeast grown at 37°C and that of yeast grown at 37°C + ptKQE remained highly correlated. In line with what previously reported (59), the proteins with the most significant differences in abundance between these two datasets are enriched for targets of the transcription factor GCN4 (Supplementary Table S5). s^2^U_34_ residues can be further modified by the ELP pathway to generate mcm^5^s^2^U_34_ (25,42). Both modifications are very similar from a phenotypical and functional point of view (25,48,60). Although a cross-talk between the pathways has been reported (25,42), we found that the activity of the ELP pathway is unaffected by growth at elevated temperatures. This might be explained by the synthetic lethality of the two pathways (25,61).

Finally, our data indicate that a number of enzymes involved in other post-transcriptional modifications of tRNAs and rRNAs are differentially abundant in yeast grown at elevated temperatures (Table 3 and Supplementary Table S2). This suggests that control of translation via post-transcriptional modification of components of the translational machinery might play a wider role in the HSR.

**Table 3.** Putative tRNA modifying enzymes with differential abundance at 30°C versus 37°C

Systematic Name	log_2_(30°C/37°C)	Adj. *p*-value	Standard Name
YGL211W	2.01	0.0001	NCS6
YKR056W	1.14	0.0001	TRM2
YOR243C	1.08	0.0002	PUS7
YFL001W	0.69	0.0006	DEG1
YNL292W	0.69	0.0006	PUS4
YML014W	−0.56	0.0011	TRM9
YHR111W	−0.58	0.0012	UBA4
YNL119W	0.69	0.0020	NCS2
YCL017C	−0.43	0.0024	NFS1
YPL207W	0.78	0.0031	TYW1
YPL212C	0.36	0.0040	PUS1
YBL024W	0.31	0.0060	NCL1
YML080W	0.41	0.0078	DUS1
YKL027W	−0.36	0.0079	YKL027W
YGL050W	−0.37	0.0083	TYW3

Protein involved in tRNA post-transcriptional modification with differential abundance (1% FDR) at 30°C versus 37°C.

In conclusion, we have shown that the temperature induced down-regulation of tRNA thiolation contributes to the HSR by reducing anabolic processes. Specifically, the diminished activity of the URM1 pathway impacts on protein translation by decreasing the translation efficiency of transcripts biased in the content of codons recognized by thiolated tRNAs. Many ribosomal, and ribosome biogenesis proteins, belong to this category, leading to a further decrease in overall protein translation. Given that the increase in thermal energy during a heat shock can promote protein aggregation, we propose that a reduction in protein synthesis rates affords proteins additional time to reach a fully folded native state and assists in maintaining proteome homeostasis.

## SUPPLEMENTARY DATA

Supplementary Data are available at NAR Online.

SUPPLEMENTARY DATA
