# Supplementary material for: Protein degradation and dynamic tRNA thiolation fine-tune translation at elevated temperatures
Source: Nucleic Acids Res. 2015 Apr 13;43(9):4701–12. doi: 10.1093/nar/gkv322 (PMC4482078; doi:10.1093/nar/gkv322)
Supplement: SUPPLEMENTARY DATA [file supp_43_9_4701__index.html]

Protein degradation and dynamic tRNA thiolation fine-tune translation at elevated temperatures — SUPPLEMENTARY DATA 

# Protein degradation and dynamic tRNA thiolation fine-tune translation at elevated temperatures

## SUPPLEMENTARY DATA

**Files in this Data Supplement:**

- SUPPLEMENTARY DATA
- SUPPLEMENTARY DATA
