# Supplementary material for: Protein degradation and dynamic tRNA thiolation fine-tune translation at elevated temperatures
Source: Nucleic Acids Res. 2015 Apr 13;43(9):4701–12. doi: 10.1093/nar/gkv322 (PMC4482078; doi:10.1093/nar/gkv322)

SUPPLEMENTARY DATA

SUPPLEMENTARY MATERIAL AND METHODS

Yeast culture and media

YPD medium was composed of 10 g/l Bacto yeast extract (Becton Dickinson, USA), 20 g/l Bacto peptone (Becton Dickinson, USA) and 2% (w/v) D-glucose. Synthetic complete (SC+D) media was composed of 1.7 g/l Difco yeast nitrogen base without amino acids and $(NH_4)_2SO_4$ (Becton Dickinson, USA), 5.0 g/l $(NH_4)_2SO_4$ (Sigma), 0.03 g/l isoleucine, 0.15 g/l valine, 0.04 g/l adenine, 0.02 g/l histidine, 0.1 g/l leucine, 0.02 g/l methionine, 0.05 g/l phenylalanine, 0.2 g/l threonine, 0.04 g/l tryptophan, 0.03 g/l tyrosine, 0.02 g/l uracil, 0.1 g/l glutamic acid, 0.1 g/l aspartic acid, 0.02 mg/ml arginine, 0.03 mg/ml lysine (all amino acids were bought from Sigma), 2% (w/v) glucose). All yeast cultures were grown to mid-log phase and for at least four doublings. Similarly, in the case of heat-stress treatment, all cultures were exposed to 37 °C for at least four doublings unless stated otherwise.

SILAC labeling

SILAC strains were exponentially grown in SC-K-R+D supplemented with 0.2 g/l proline and 0.02 mg/ml arginine, 0.03 mg/ml lysine or 0.02 mg/ml $^{13}C_6$,$^{15}N_4$-arginine (Sigma), 0.03 mg/ml $^{13}C_6$,$^{15}N_2$-lysine (Sigma). The precultures were also grown in the labelled medium and cells were grown for at least 4 doublings in the main culture to ensure complete labelling. After harvesting, equal OD_{600} of light and heavy cultures were mixed and subsequently processed together.

Protein extraction and digestion for MS

After harvesting yeast cells were treated with 1.85 M NaOH, 7.6% (v/v) β-mercaptoethanol for 10 min on ice then with an equal volume of 50% (w/v) trichloroacetic acid (CCl_3COOH) for 20 min on ice. The precipitated proteins were pelleted and washed with acetone at least three times. Tryptic digestion was carried out using the FASP method (1) with the sequencing grade modified trypsin (V5113, Promega). Digested peptides were purified on C18 MacroSpin columns (The Nest Group).

Peptide fractionation by isoelectric focusing (OffGel)

C18 purified peptides were fractionated in 12 fractions by isoelectric focusing on an OffGel Fractionator (G3100AA, Agilent) as per manufacturer instructions, except that the strips used were pH 3-11 NL, 13 cm (17-6003-75, GE Healthcare) and the ampholytes were 2% IPG buffer pH 3-11 NL (17-6004-40, GE Healthcare).

LC-MS-MS/MS

C18 cleaned peptides were resuspended in 0.1% CF_3COOH for the LC-MS-MS/MS analysis. An Ultimate3000 uHPLC system (Dionex, Thermo Scientific) was used for the online reverse phase liquid chromatography. Coated-tip fused silica columns of 50 cm length (PF-360-75-10-CE-5, New Objective) were in-house packed with C18 silica beads (Magic C18, 200 Å, 3 µm, Michrom Bioresources). A 250 nl/min gradient of buffer B (0.1% (v/v) HCOOH, 90% (v/v) CH_3CN, 3% (v/v) DMSO) in buffer A (0.1% (v/v) HCOOH, 2% (v/v) CH_3CN, 3% (v/v) DMSO) ranging from 2% to 35% over 240 min for the OffGel fractionated samples or for 360 min for unfractionated samples was used to resolve peptides. The column oven compartment was maintained at 45 °C. The chromatography setup was directly coupled to an LTQ-Orbitrap Velos Pro mass spectrometer (Thermo Finnigan) configured for top-15 data dependent acquisition (DDA) by collision-induced fragmentation (CID). MS1 scans were done in profile mode at the FT-MS resolution of 60,000 and MS/MS scans were done in centroid mode with the IonTrap Rapid Scan Rate. The precursor ion intensity threshold for

triggering fragmentation was set at 500 arbitrary units. Dynamic exclusion was enabled with a repeat count set at 1, repeat duration at 30 s, exclusion list size of 500 and exclusion duration of 90 s. Preview mode for FT-MS master scans, monoisotopic precursor selection and charge state screening with +1 rejection were enabled.

Protein identification and quantification

MS data were processed using the Trans-proteomic pipeline (2). Raw files were converted to the mzXML file format (3) and searched using Comet (4) against the Saccharomyces Genome Database protein database. For the searches, carboxyamidomethylation (57.022 Da) of Cys was set as a static modification and $^{13}C_6,^{15}N_2$-Lys (8.01419892 Da), $^{13}C_6,^{15}N_4$-Arg (10.008252778 Da) and oxidation of Met (15.99491463 Da) were set as variable modifications. Semi-tryptic digestion was set with a maximum of two missed cleavages. The mass error tolerances used were 25 ppm and 0.4 Da for MS and MS/MS, respectively. PeptideProphet and ProteinProphet were used for the evaluation of peptide and protein probabilities, respectively (5, 6). Protein abundance ratios were estimated by XPRESS (7). Results were stored in an in-house developed database.

Data normalisation and statistical analysis of differential abundance

Protein and peptide abundance ratios were imported into R. Peptides not passing the 1% FDR threshold were removed and the remaining peptide ratios were median normalised. Next, the peptides that were consistently quantified across label switch conditions were used to calculate protein abundance ratios. Subsequently, proteins meeting 1% FDR and having at least two quantified peptides were retained and protein abundance ratios were median normalised. Finally, statistical significance of changes in protein abundance was calculated using an empirically moderated Bayes t-test and *p*-values were adjusted for multiple testing by Benjamini and Hochberg procedure. The FDR threshold used for statistical significance of protein abundance ratios was 1% (adjusted *p*-value = 0.01).

Random Forest analysis for codon importance

Random Forest analyses of the codon content for the mRNAs corresponding to significantly changing proteins were done in R using the random forest implementation of the party package (v0.9- 99992). The abundance of all codons except the stop ones were used as variables in the analysis. The number of trees in the forest was 1,000 and the number of predictor variables was set to square root of the total number of variables.

Quantitative PCR

Ribo-pure yeast kit (AM1926, Life Technologies) was used to extract the total RNA from exponentially growing yeast cells. The cDNA library was prepared using the High Capacity cDNA Reverse Transcription Kit with RNase Inhibitor (4374966, Life Technologies) according to the manufacturer's protocols. Estimation of the mRNA levels was done using the quantitative real time PCR with the predesigned TaqMan gene expression assays on an Applied Biosystems 7900HT Fast Real-Time PCR System.

Western blotting

Exponentially growing yeast cells were harvested by centrifugation and lysed by treatment with 1.85 M NaOH, 7.6% β-mercaptoethanol for 10 min on ice followed by protein precipitation with 50% (w/v) TCA for 15 min on ice. Precipitated protein pellets were washed with acetone, air-dried and resolubilised in 10% (w/v) SDS, 1.0 M unbuffered Tris and reduced with an equal volume of 0.2 M DTT, 30% (v/v) glycerol, 0.002% (w/v) bromophenol blue for 30 min at 65 °C. The denatured protein samples were separated by poly-acrylamide gel electrophoresis, transferred onto a nitrocellulose membrane and probed with specific antibodies (mouse-anti-HA antibody (HA.11 Clone 16B12; MMS-101R, Covance), mouse-

anti-Actin antibody (ab8224, Abcam) and goat-anti-mouse IgG, IgA, IgM (H+L) horseradish peroxidase conjugate (A-10668, Invitrogen)) using standard procedures.

Extraction of total RNA and enrichment for bulk tRNA

For small scale extraction, 25 ml of an exponentially growing yeast culture was harvested by centrifugation and resuspended in 0.5 ml of 150 mM NaCl solution. The cells were lysed by bead beating (G8772, Sigma Aldrich) in 750 μl saturated phenol for 7 min. Subsequently, 75 μl of chloroform were added and the mixture was vortexed for 1 min. After centrifugation the aqueous phase was transferred to a new tube and re-purified with 500 μl phenol and 50 μl chloroform. The tubes with organic phases were re-extracted with 2 ml 150 mM NaCl. The aqueous phases were pooled and loaded into a Nuclebond AX20 anion exchange columns (Macherey-Nagel, 740511). Prior to sample loading, the columns were equilibrated with 3 ml of column buffer (10 mM Tris pH 6.8, 15% (v/v) Ethanol, 200 mM KCl). The bound RNA was washed with 6.0 ml of column buffer. tRNA was eluted with 1.5 ml elution buffer (10 mM Tris pH 6.5, 15% Ethanol, 650 mM KCl) and concentrated by precipitation with 2.5x volumes of Ethanol at –20 °C overnight. The precipitated tRNA was washed extensively in 80% (v/v) ice-cold ethanol and air-dried. The tRNA pellet was resuspended in nuclease free water (Applied Biosystems, AM9930). For large scale applications, 1 litre of yeast culture was grown and the volume of the various reagents used was scaled proportionally. Nucleobond AX500 columns were used for the enrichment of bulk tRNA

Purification of specific tRNAs

3'-biotinylated DNA olginucleotide probes, specific for the amino-acyl arm of the tRNAs to be purified, were purchased from Invitrogen. Streptavidin-sepharose resin (17-5113-01, GE Healthcare) was used at an approximate ratio of 1 nmole of probe to 10 μl of the resin slurry. Prior to coupling, the streptavidin beads were extensively washed at room temperature; first in coupling buffer (5 mM Tris-HCL (pH 7.5), 1 mM EDTA (pH 8.0), 1 M NaCl) for 10 min, then twice in 0.1 M NaOH, 0.05 M NaCl for 2 min each, then once in 0.1 M NaCl solution for 2 min and finally, twice in coupling buffer for 5 min each. Following the washes, the probe was added to the resin in coupling buffer and incubated for 45 min at 37 °C on a thermo-mixer. The coupled resin was washed once with coupling buffer and 4 times with tRNA binding buffer (30 mM HEPES-KOH (pH 7.5), 15 mM EDTA (pH 8.0), 1.2 M NaCl). About 400 µg of bulk tRNA dissolved in tRNA binding buffer was added and the mixture was incubated at 65 °C for 15-20 min to partially denature the tRNAs. Then, the mixture was moved to a rotating-wheel for 1 h at room temperature to allow annealing of the tRNAs with immobilised probes. Subsequently, the resin was washed 5 times with tRNA wash buffer (100 mM NaCl, 2.5 mM HEPES-KOH (pH 7.5), 1.25 mM EDTA (pH 8.0)) at 37 °C for 5 min each. Finally, the tRNAs were eluted twice in elution buffer (20 mM NaCl, 0.5 mM HEPES-KOH (pH 7.5), 0.25 mM EDTA (pH 8.0)) at 70 °C for 15 min each. The eluted tRNAs were precipitated overnight at –20 °C with 0.1 volumes of 20% (w/v) NaOAc and 2.5x volumes of ethanol.

The probes used were; 5'-CTCCGATACGGGGAGTCGAACCCCGGTCTC-3' for Sc-tE^{UUC},
5'-AGGTCCTACCCGGATTCGAACCGGGGTTGT-3' for Sc-tQ^{UUG} and
5'-CACTCACGATGGGGGTCGAACCCATAATCT-3' for Sc-tR^{UCU}.

RNA mass spectrometry analysis

10 μg of a specific tRNA were digested and dephosphorylated in a 50 µl reaction volume with 2U of Nuclease P1 (N7000-USB, Stratech), 300U of bacterial alkaline phosphatase (18011-015, Life Technologies) in the presence of 0.9 mM $ZnSO_4$ at 37 °C for 90-120 min. 15 µl of 0.5 M NH_4HCO_3 was added and incubated at 37 °C for 90-120 min. The reaction mixture was acidified using HCOOH, the nucleosides were purified over graphite TopTips (Glygen Corp.) and dried in a vacuum dryer.

For the mass spectrometry analysis, the nucleosides were resuspended in water and separated by liquid chromatography over an Hypercarb graphite column (35005-100065, Thermo Scientific) connected to an UltiMate 3000 uHPLC system (Dionex) running a 1.0 µl/min gradient of buffer B (0.1% (v/v) HCOOH, 90% (v/v) CH_3CN) in buffer A (0.1% (v/v) HCOOH, 2% (v/v) CH_3CN) ranging from 1% to 37% over 45 min. The column-oven temperature was maintained at 65 °C throughout the run. A fused silica emitter (PicoFrit columns, PF360-75-10-N-5, New Objective) was used to spray eluting nucleosides into an LTQ-Orbitrap Velos Pro mass spectrometer (Thermo Finnigan) operating at a source voltage of 1.8 kV. MS1 scans were acquired in profile mode at an FT-MS resolution of 100,000. The MS was running in data-independent acquisition mode (DIA) using collision-induced fragmentation (CID) with an isolation width set at 2.0 m/z and normalised collision energy of 35.0. MS/MS spectra were acquired in centroid mode with the IonTrap Normal scan rate. Raw MS files were converted to the mzXML format (3) and analyzed by an in-house developed software to identify and quantify the nucleosides. The identifications were validated by manually comparing the fragmentation spectra with previously reported ones (9, 10).

APM supplemented denaturing PAGE

0.5 to 1.0 µg of bulk tRNA was mixed with an equal volume of loading buffer (AM8547, LifeTechnologies) and electrophoresed through a 10% acrylamide gel containing 7 M urea, 0.5X TBE and 50 µg/ml [(N- acryloylamino)phenyl] mercuric chloride (APM) (synthesised in house according to the procedure described in (11) at 200 V for 90 min. The gels were pre-run for 15 minutes at 200 V and each well was washed by pipetting electrophoresis buffer multiple times. After electrophoresis, the gels were stained with SYBR Gold (Invitrogen) diluted 1:10,000 in 0.5X TBE for 5 min before imaging.

tRNA northern blot analysis

Electrophoretically separated tRNAs were transferred to a positively charged nylon membrane (Hybond-N+, GE Healthcare) using a semi-dry blotter (Fastblot, biometra) at a constant current of 400 mA for 40 min in 0.5X TBE as the transfer buffer. Transferred tRNAs were cross linked using a UV crosslinker (C-1000 UVP) at an energy setting of 1200 J for 30 s, while the membrane was still moist. Subsequently, the membrane was prehybridised using 5ml of PerfectHyb Plus (Sigma) buffer with 1x ProtectRNA RNAse inhibitor and 0.1 *µ*g/*µ*l ssDNA for 2 h in a 50 ml centrifuge tube. 10 pmol of oligonucleotide probe was labeled with 10U of T4 Polynucleotide Kinase (New England Biolabs) and 5 *µ*l of 3000 Ci/mmol 10 uCi/*µ*l ϒ ^{32}P ATP (PerkinElmer) at 37 °C for 60 min. Labelling was quenched by 1 *µ*l of 0.5 M EDTA at 65 °C for 20 min. The labeled probes were cleaned using the illustra MicroSpin G-25 Columns (GE Healthcare). After pre-hybridisation the labeled probe was added directly to the tubes and incubated overnight at 55 °C in a hybridisation oven. The membrane was washed twice for 10 min each and again twice for 30 min each at 55 °C using 10 ml wash buffer (3x SSC, 25 mM NaH_2PO_4 pH 7.5, 5% SDS, 10x Denhardt's reagent) pre-warmed to 37 °C. Finally, the membrane was washed for 8 min in high stringency buffer (1x SSC, 10% SDS) pre-warmed to 37 °C and exposed to the X-Ray Film.

The probes used were; 5'-CTCCTCATAGGGGGCTCGAACCC-3' for Sc-tK^{UUU},
5'-AGGTCCTACCCGGATTCGAACCGG-3' for Sc-tQ^{UUG},
5'-CGCCCAAACAGGGACTTGAACCC-3' for Hs-tK^{UUU},
5'-GGTCCCACCGAGATTTGAACTCGG-3' for Hs-tQ^{UUG},
5'-CGACTCTGGTGGGATTCGAACCC-3' for Hs-tR^{UCU} and
5'-TGCGTTGGCCGGGAATCGAACCCG-3' for Hs-tG^{UCC}.

SUPPLEMENTARY DATA REFERENCES

1. Wiśniewski, J.R., Zougman, A., Nagaraj, N. and Mann, M. (2009) Universal sample preparation method for proteome analysis. *Nat Methods*, **6**, 359–362.
2. Pedrioli, P.G.A. (2010) Trans-proteomic pipeline: a pipeline for proteomic analysis. In Hubbard, S.J., Jones, A.R. (eds), *Proteome Bioinformatics, Methods in Molecular Biology*. Humana Press, Totowa, NJ, Vol. 604, pp. 213–238.
3. Pedrioli, P.G.A., Eng, J.K., Hubley, R., Vogelzang, M., Deutsch, E.W., Raught, B., Pratt, B., Nilsson, E., Angeletti, R.H., Apweiler, R., et al. (2004) A common open representation of mass spectrometry data and its application to proteomics research. *Nature biotechnology*, **22**, 1459–1466.
4. Eng, J.K., Jahan, T.A. and Hoopmann, M.R. (2012) Comet: An open-source MS/MS sequence database search tool. *Proteomics*, **13**, 22–24.
5. Nesvizhskii, A., Keller, A., Kolker, E. and Aebersold, R. (2003) A statistical model for identifying proteins by tandem mass spectrometry. *Anal Chem*, **75**, 4646–4658.
6. Keller, A., Nesvizhskii, A., Kolker, E. and Aebersold, R. (2002) Empirical statistical model to estimate the accuracy of peptide identifications made by MS/MS and database search. *Anal Chem*, **74**, 5383–5392.
7. Han, D.K., Eng, J., Zhou, H. and Aebersold, R. (2001) Quantitative profiling of differentiation-induced microsomal proteins using isotope-coded affinity tags and mass spectrometry. *Nature biotechnology*, **19**, 946–951.
8. Rezgui, V.A.N., Tyagi, K., Ranjan, N., Konevega, A.L., Mittelstaet, J., Rodnina, M.V., Peter, M. and Pedrioli, P.G.A. (2013) tRNA tKUUU, tQUUG, and tEUUC wobble position modifications fine-tune protein translation by promoting ribosome A-site binding. *Proceedings of the National Academy of Sciences*, **110**, 12289–12294.
9. Leidel, S., Pedrioli, P.G.A., Bucher, T., Brost, R., Costanzo, M., Schmidt, A., Aebersold, R., Boone, C., Hofmann, K. and Peter, M. (2009) Ubiquitin-related modifier Urm1 acts as a sulphur carrier in thiolation of eukaryotic transfer RNA. *Nature*, **458**, 228–232.
10. Bullinger, D., Fux, R., Nicholson, G., Plontke, S., Belka, C., Laufer, S., Gleiter, C.H. and Kammerer, B. (2008) Identification of urinary modified nucleosides and ribosylated metabolites in humans via combined ESI-FTICR MS and ESI-IT MS analysis. *J. Am. Soc. Mass Spectrom.*, **19**, 1500–1513.
11. Igloi, G. (1988) Interaction of tRNAs and of phosphorothioate-substituted nucleic acids with an organomercurial. Probing the chemical environment of thiolated residues by affinity …. *Biochemistry*.
12. Brachmann, C., Davies, A., Cost, G., Caputo, E., Li, J., Hieter, P. and Boeke, J. (1998) Designer deletion strains derived from Saccharomyces cerevisiae S288C: a useful set of strains and plasmids for PCR-mediated gene disruption and other applications. *Yeast*, **14**, 115–132.
13. Ghislain, M., Udvardy, A. and Mann, C. (1993) S. cerevisiae 26S protease mutants arrest cell division in G2/metaphase. *Nature*, **366**, 358–362.
14. Ghaemmaghami, S., Huh, W.-K., Bower, K., Howson, R.W., Belle, A., Dephoure, N., O'Shea, E.K. and Weissman, J.S. (2003) Global analysis of protein expression in yeast. *Nat Cell Biol*, **425**, 737–741.
15. Nagaraj, N., Kulak, N.A., Cox, J., Neuhauser, N., Mayr, K., Hoerning, O., Vorm, O. and Mann, M. (2012) System-wide perturbation analysis with nearly complete coverage of the yeast proteome by single-shot ultra HPLC runs on a bench top Orbitrap. *Mol Cell Proteomics*, **11**, M111.013722.
16. Gasch, A.P., Spellman, P.T., Kao, C.M., Carmel-Harel, O., Eisen, M.B., Storz, G., Botstein, D. and Brown, P.O. (2000) Genomic expression programs in the response of yeast cells to environmental changes. *Molecular Biology of the Cell*, **11**, 4241–4257.
17. Robinson, M.D., Grigull, J., Mohammad, N. and Hughes, T.R. (2002) FunSpec: a web-based cluster interpreter for yeast. *Bmc Bioinformatics*, **3**, 35.

SUPPLEMENTARY FIGURE LEGENDS

Supplementary Figure S1: Filtering and quality control of the 30 °C vs. 37 °C comparison. The SILAC labelling design for replicate number 2, was switched (denoted by label design -1). The means of $\log_2$ of ratios of each peptide from the two designs were compared and the peptides that were not

consistently quantified across the label switch conditions were removed. (A) and (B) represent the distributions of peptide ratios before and after the label switch filter in the form of a scatter plot of binned hexagonal tiles. The tiles colour indicates the number of peptides in each bin. (C) MA plots for the three biological replicates. M: log_2(peptide ratio); A: (log_2(light peptide area) + log_2(heavy peptide area)) / 2. (D) and (E) Box-and-Whisker plots of the protein ratios from the three biological replicates before and after median normalisation respectively. The box represents the inter quartile range (50% of the data) with the solid-line in the centre of the box representing the median of the distribution of protein ratios. (F) Dynamic range of protein expression after stringent filtering. The histogram illustrates the number of quantified proteins passing the filtering criteria in relation to their copy per cell number (14). The red and blue dashed lines represent the median and mean in the linear scale, respectively.

Supplementary Figure S2: 30 °C vs 37 °C proteomics data quality check and normalisation. (A) Scatter plot of the statistically significant protein ratios in common in the present study and the heat shock response study by Nagaraj et al. (15). (B) Heat-map comparison of statistically significant changes in protein abundances from this study and in mRNA abundances estimated from Gasch et al. (16). Micro-array analyses ct1 29 °C/37 °C along with two repeats of ct2 37 °C vs. 29 °C were treated as replicates and a Bayes moderated *t*-test was used to determine significant changes. Only significant changes in common are plotted. (C - D) Word clouds showing the most significantly over-represented (*p*-value < 0.001) GO terms for Biological Processes from (C) up- or (D) down-regulated proteins; analysed by FunSpec (17). GO terms in common with those significantly over-represented in the wild-type vs. *urm1Δ* analysis (presented in (8)) are shown in red.

Supplementary Figure S3: Manual validation of Urm1p identification and quantification. (A) and (B) are the annotated MS/MS spectra for the heavy and light versions of the only Urm1p peptide identified and quantified in the wt 30 °C/37 °C proteomics analysis. The annotated spectra were obtained from the Lorikeet Spectrum Viewer for Comet (available as part of the TPP). (C) and (D) are the extracted ion chromatograms obtained from XPRESS for the light and heavy Urm1p peptide. The abundance of Urm1p at 30 °C and 37 °C is not significantly different as judged by one-sample *t*-test (*p*-value = 0.39).

Supplementary Figure S4: Manual validation of Ncs2p identification and quantification. Shown are the annotated MS/MS spectra, obtained from the Lorikeet Spectrum Viewer for Comet, and the extracted ion chromatograms (XICs), obtained from XPRESS, for the indicated peptides of Ncs2p that were identified and quantified in the WT 30 °C/37 °C proteomics analysis. Refer to Table 2 and Supplementary Table S2 for the mean Ncs2p protein abundance ratio and the associated statistical significance.

Supplementary Figure S5: Manual validation of Ncs6p identification and quantification. Shown are the annotated MS/MS spectra, obtained from the Lorikeet Spectrum Viewer for Comet, and the extracted ion chromatograms (XICs), obtained from XPRESS, for the indicated peptides of Ncs6p that were identified and quantified in the WT 30 °C/37 °C proteomics analysis. Refer to Table 2 and Supplementary Table S2 for the mean Ncs6p protein abundance ratio and the associated statistical significance.

Supplementary Figure S6: tRNA hypothiolation and crosstalk between the URM1 and ELP pathways. (A) WT cells, growing exponentially at 30 °C, were diluted into prewarmed YPD medium and incubated at 37 °C. Samples were taken at the indicated time points, processed to extract bulk tRNA and analysed by [p-(N-acrylamino)-phenyl]mercuric chloride (APM) supplemented denaturing PAGE. ON: over-night. (B) Electropherogram of bulk tRNA isolated from wild-type, *urm1Δ* or *elp1Δ* cells and analysed by APM-dPAGE. APM interacts and retards the thiolated tRNAs that appear as a slow moving band at the top of the gel. (C) tE^{UUC} runs as a single band in a PAGE after purification.

Supplementary Figure S7: MS and MS/MS spectra for mcm^5U and mcm^5s^2U. (A) MS spectra of mcm^5s^2U and mcm^5U nucleoside and nucleobase are shown. (B) MS/MS spectra of mcm^5s^2U and mcm^5U nucleobases obtained after CID based fragmentation. Spectra were annotated by matching against the published fragmentation products of the modified uridines (9,10).

Supplementary Figures S8, S9 and S10: Extracted ion chromatograms for mcm^5U and mcm^5s^2U. tRNA tQ^{UUG} (S8), tE^{UUC} (S9) and tR^{UCU} (S10) were purified from wild-type yeast cells grown in either rich medium at 30 °C, or rich medium at 37 °C, or in absence of sulfur amino acids at 30 °C, and were digested and dephosphorylated to nucleosides and analysed by LC-MS-MS/MS. Shown here are the extracted ion chromatograms (XICs) for the m/z values corresponding to protonated nucleoside (MH^+) and nucleobase (BH^+) of A, mcm^5s^2U and mcm^5U from either tQ^{UUG}, tE^{UUC} or tR^{UCU}. The area under the XIC was used to estimate the abundance of the corresponding nucleoside. Note that the overlapping nucleoside-nucleobase elution profiles are generated by post-source fragmentation of the N-glycosidic bond. This overlap is used as a criterion for the identification of a nucleoside in addition to its MS/MS spectra (9).

Supplementary Figure S11: Volcano plots for codon biases. Each volcano plot in the figure shows the abundance ratio and statistical significance of the proteins whose corresponding genes are among the top 2% of yeast genes with the highest frequency of the indicated codon. The dotted red line (y=2) indicates 1% FDR. The black dotted line (x=0) indicates a protein ratio of 1.

Supplementary Figure S12: The tRNA recognising AGA is thiolated in higher eukaryotes. APM-dPAGE and northern blot analyses of the bulk tRNAs isolated from HEK-293 cells.

Supplementary Figure S13: An *NCS2* mutant deregulates tRNA thiolation. Electropherogram from APM-dPAGE analysis of the bulk tRNA isolated from wild-type (WT) or *ncs2Δ* cells transformed with either an empty plasmid (pEV), a plasmid with wild-type NCS2 (pNCS2), or one with *ncs2_A212T* (p*ncs2_A212T*) and grown under the indicated conditions.

Supplementary Figure S14: Over-expression of tRNAs does not affect their thiolation. Electropherogram from APM-dPAGE analysis of bulk tRNA isolated from wild-type (WT) cells transformed with either a high-copy empty plasmid (pEV) or the same plasmid with tRNA genes for tK^{UUU}, tQ^{UUG} and tE^{UUC} (ptKQE) and grown under the indicated conditions.

Supplementary Figure S15: Filtering and quality control of the 30 °C vs 37 °C + ptKQE analysis. SILAC labelling design for replicate number 2, was switched (denoted by label design -1). The means of $\log_2$ of ratios of each peptide from the two designs were compared and the peptides that were not consistently quantified across the label switch conditions were removed. (A) and (B) represent the distributions of peptide ratios before and after the label switch filter in the form of a scatter plot of binned hexagonal tiles. The tiles colour indicates the number of peptides in each bin. (C) Peptide level MA plots. M is equal to the $\log_2$ of peptide abundance ratios (30 °C/37 °C + ptKQE) and A is equal to 1/2 * $\log_2$ of the product of peptide abundances from the two conditions. Systematic inter-sample variances were checked and removed using median normalisation. (D) and (E) Box-and-Whisker plots of the protein ratios from the three biological replicates before and after median normalisation respectively. The box represents the inter quartile range (50% of the data) with the solid-line in the centre of the box representing the median of the distribution of protein ratios.

Supplementary Figure S1

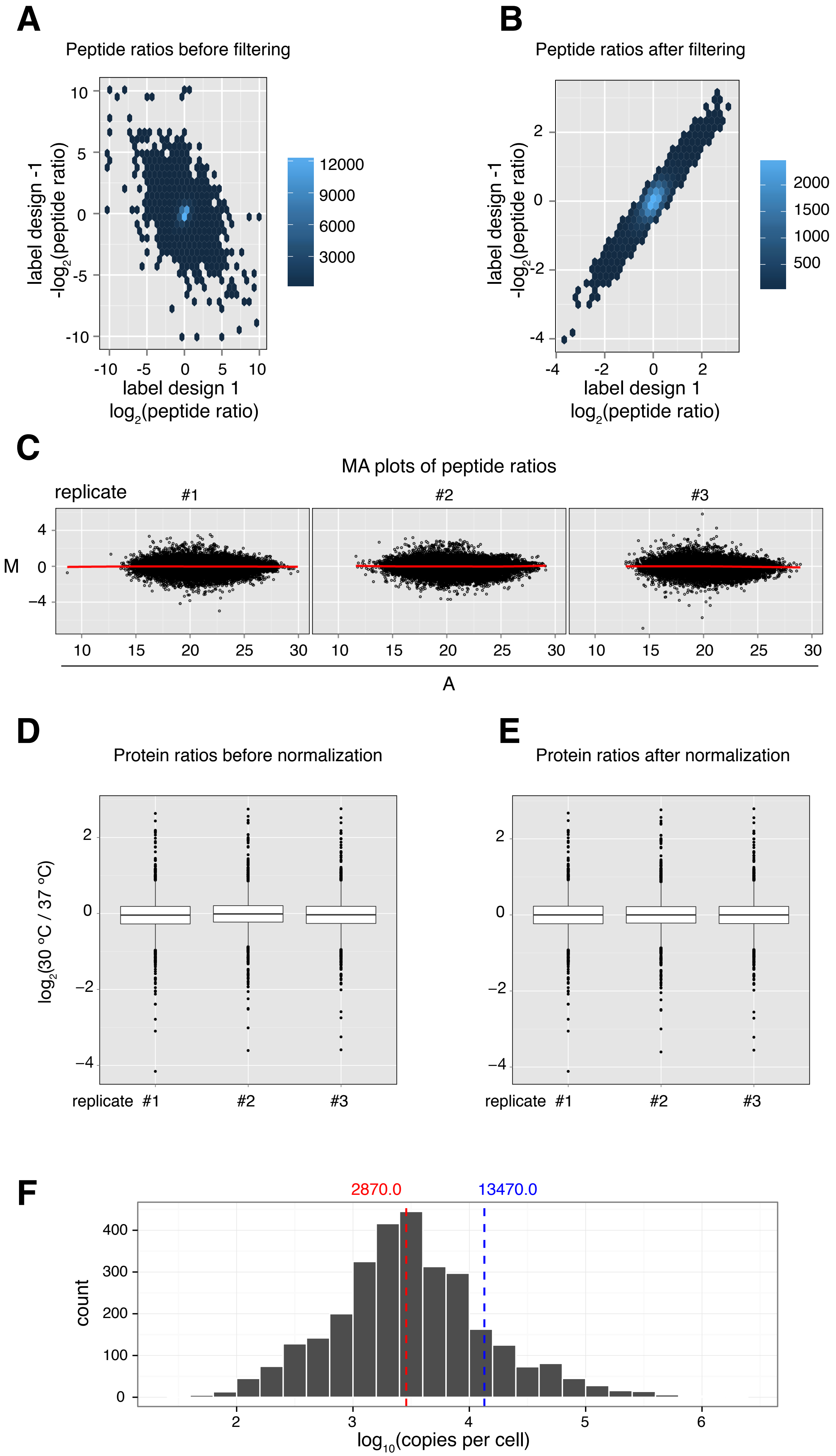

Supplementary Figure S2

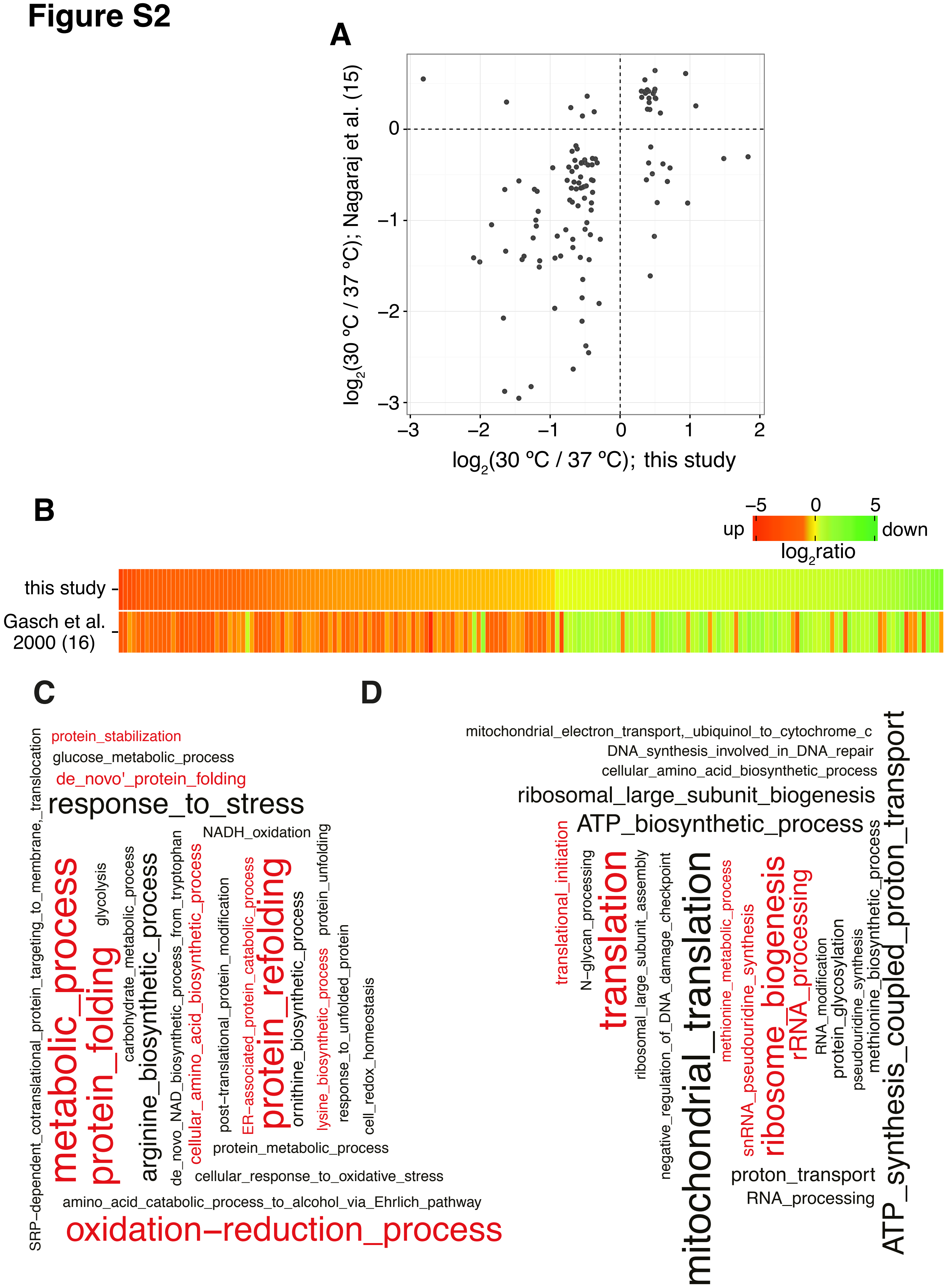

Supplementary Figure S3

A

VNVKVEFLGGLDAIFGK, MH+ 1822.0445, m/z 608.0197

Exp. m/z: 608.025, Charge: 3

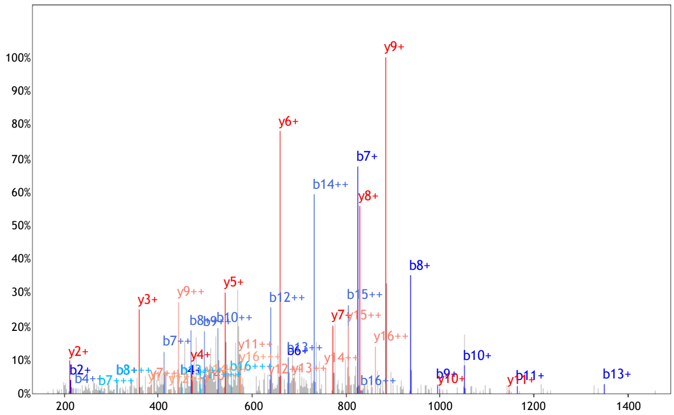

b+	b2+	b3+	#	Seq	#	y+	y2+	y3+
100.0757	50.5415	34.0301	1	V	17			
214.1186	107.5629	72.0444	2	N	16	1722.9761	861.9917	574.9969
313.1870	157.0972	105.0672	3	V	15	1608.9331	804.9702	536.9826
449.2962	225.1518	150.4369	4	K	14	1509.8647	755.4360	503.9598
548.3646	274.6860	183.4597	5	V	13	1373.7555	687.3814	458.5900
677.4072	339.2073	226.4739	6	E	12	1274.6871	637.8472	425.5672
824.4757	412.7415	275.4967	7	F	11	1145.6445	573.3259	382.5530
937.5597	469.2835	313.1914	8	L	10	998.5761	499.7917	333.5302
994.5812	497.7942	332.1986	9	G	9	885.4920	443.2497	295.8355
1051.6026	526.3050	351.2057	10	G	8	828.4706	414.7389	276.8284
1164.6867	582.8470	388.9004	11	L	7	771.4491	386.2282	257.8212
1279.7137	640.3605	427.2427	12	D	6	658.3650	329.6862	220.1265
1350.7508	675.8790	450.9218	13	A	5	543.3381	272.1727	181.7842
1463.8348	732.4211	488.6165	14	I	4	472.3010	236.6541	158.1052
1610.9032	805.9553	537.6393	15	F	3	359.2169	180.1121	120.4105
1667.9247	834.4660	556.6464	16	G	2	212.1485	106.5779	71.3877
			17	K	1	155.1270	78.0672	52.3805

B

VNVKVEFLGGLDAIFGK, MH+ 1806.0160, m/z 602.6768

Exp. m/z: 602.6833, Charge: 3

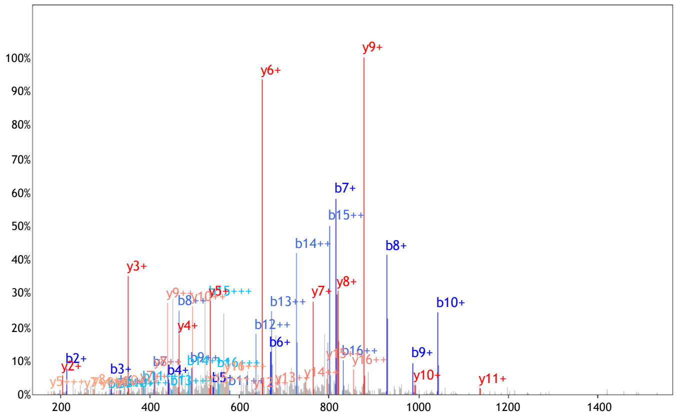

b+	b2+	b3+	#	Seq	#	y+	y2+	y3+
100.0757	50.5415	34.0301	1	V	17			
214.1186	107.5629	72.0444	2	N	16	1706.9476	853.9774	569.6540
313.1870	157.0972	105.0672	3	V	15	1592.9047	796.9560	531.6397
441.2820	221.1446	147.7655	4	K	14	1493.8362	747.4218	498.6169
540.3504	270.6788	180.7883	5	V	13	1365.7413	683.3743	455.9186
669.3930	335.2001	223.8025	6	E	12	1266.6729	633.8401	422.8958
816.4614	408.7343	272.8253	7	F	11	1137.6303	569.3188	379.8816
929.5455	465.2764	310.5200	8	L	10	990.5619	495.7846	330.8588
986.5669	493.7871	329.5272	9	G	9	877.4778	439.2425	293.1641
1043.5884	522.2978	348.5343	10	G	8	820.4563	410.7318	274.1570
1156.6725	578.8399	386.2290	11	L	7	763.4349	382.2211	255.1498
1271.6994	636.3533	424.5713	12	D	6	650.3508	325.6790	217.4551
1342.7365	671.8719	448.2504	13	A	5	535.3239	268.1656	179.1128
1455.8206	728.4139	485.9450	14	I	4	464.2867	232.6470	155.4338
1602.8890	801.9481	534.9679	15	F	3	351.2027	176.1050	117.7391
1659.9105	830.4589	553.9750	16	G	2	204.1343	102.5708	68.7163
			17	K	1	147.1128	74.0600	49.7091

C

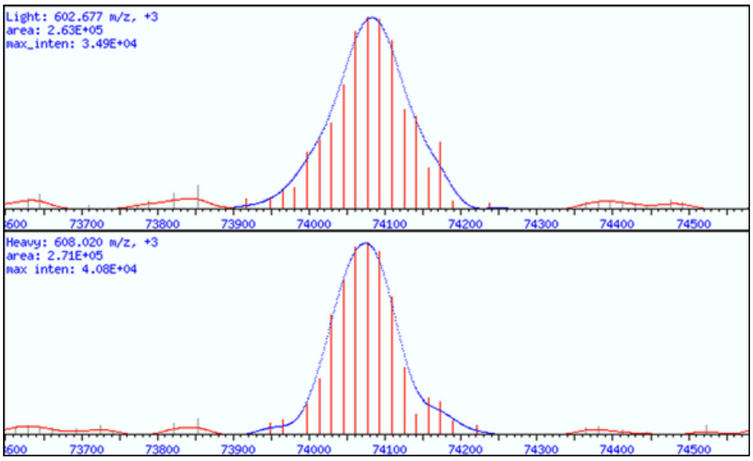

D

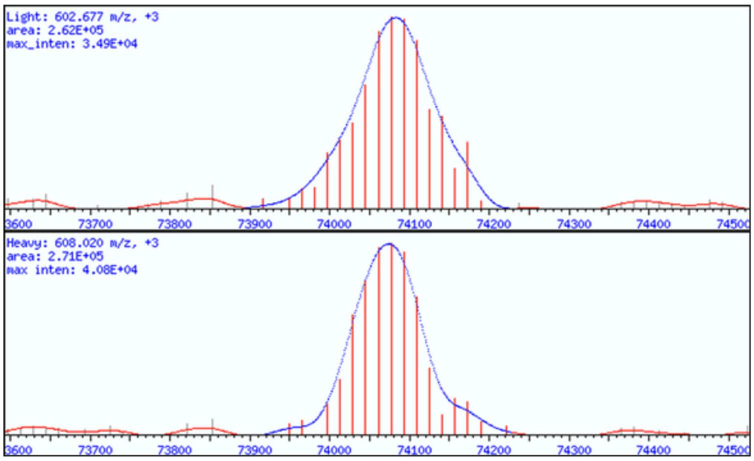

Supplementary figure S4

MS/MS and XICs for NCS2 peptide MESLINEK

sequence identified: R.MESLINEK$_{136.11}$.M
m/z = 486.2515, z = 2
ratio(30 °C/37 °C) = Heavy/Light = 1.57

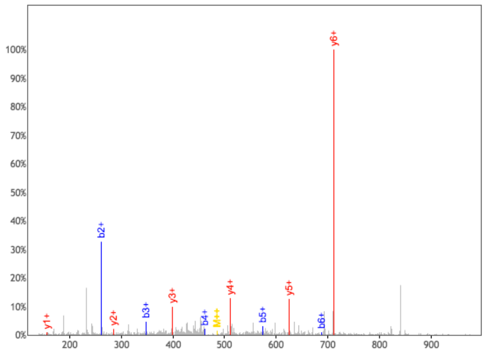

b+	#	Seq	#	y+
132.2033	1	M	8	
261.3173	2	E	7	840.9320
348.3946	3	S	6	711.8180
461.5523	4	L	5	624.7407
574.7099	5	I	4	511.5831
688.8125	6	N	3	398.4255
817.9265	7	E	2	284.3228
	8	K	1	155.2088

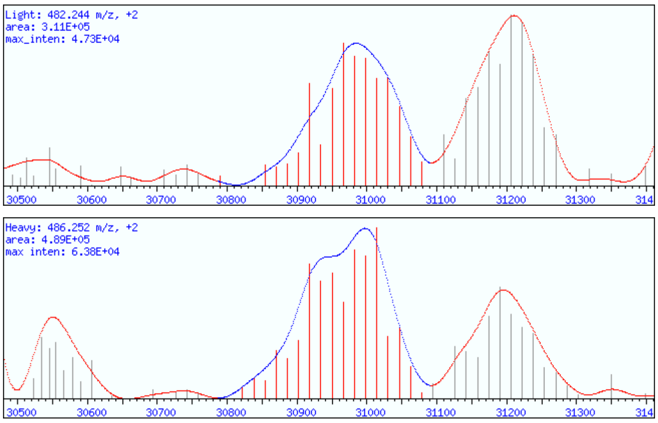

MS/MS and XICs for NCS2 peptide NLAQFLINVEETNVKPNCLIAR

sequence identified: R.NLAQFLINVEETNVK$_{136.11}$PNC$_{160.03}$LIAR$_{166.11}$.K
m/z = 858.7991, z = 3
ratio(30 °C/37 °C) = Heavy/Light = 1.97

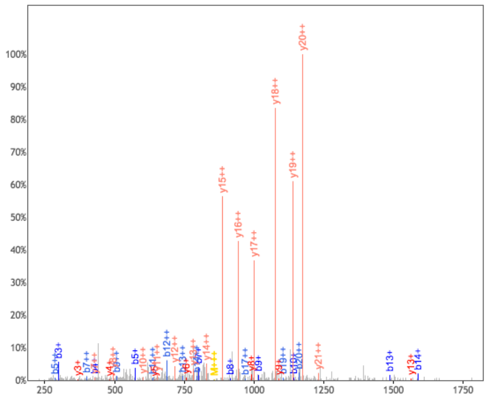

b+	b2+	#	Seq	#	y+	y2+
115.1099	58.0586	1	N	22		
228.2676	114.6374	2	L	21	2461.8313	1231.4193
299.3455	150.1764	3	A	20	2348.6736	1174.8405
427.4747	214.2410	4	Q	19	2277.5957	1139.3015
574.6485	287.8279	5	F	18	2149.4665	1075.2369
687.8062	344.4067	6	L	17	2002.2926	1001.6500
800.9638	400.9855	7	I	16	1889.1350	945.0711
915.0665	458.0369	8	N	15	1775.9774	888.4923
1014.1975	507.6024	9	V	14	1661.8747	831.4410
1143.3115	572.1594	10	E	13	1562.7437	781.8755
1272.4255	636.7164	11	E	12	1433.6297	717.3185
1373.5294	687.2683	12	T	11	1304.5157	652.7615
1487.6320	744.3196	13	N	10	1203.4118	602.2096
1586.7631	793.8852	14	V	9	1089.3092	545.1582
1722.9496	861.9784	15	K	8	990.1781	495.5927
1820.0648	910.5360	16	P	7	853.9916	427.4994
1934.1674	967.5873	17	N	6	756.8764	378.9419
2094.3317	1047.6695	18	C	5	642.7738	321.8905
2207.4893	1104.2483	19	L	4	482.6095	241.8084
2320.6470	1160.8271	20	I	3	369.4518	185.2296
2391.7249	1196.3661	21	A	2	256.2942	128.6507
		22	R	1	185.2163	93.1118

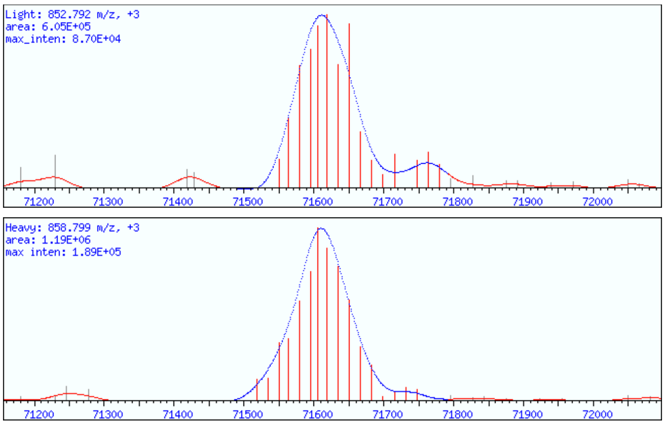

Supplementary figure S5

MS/MS and XICs for NCS6 peptide CGYLSSNNICK

sequence identified: R.$C_{160.03}$GYLSSNNI$C_{160.03}$$K_{136.11}$.A

m/z = 662.2992, z = 2

ratio(30 °C/37 °C) = Heavy/Light = 4.31

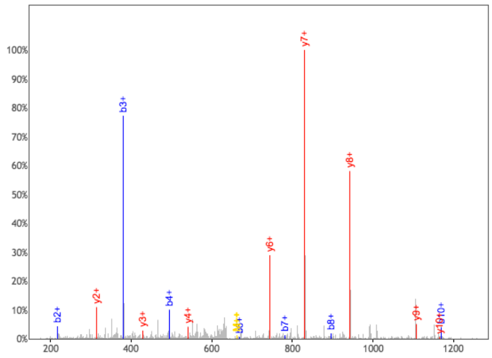

b+	#	Seq	#	y+
161.1716	1	C	11	
218.2229	2	G	10	1164.2729
381.3962	3	Y	9	1107.2216
494.5538	4	L	8	944.0483
581.6311	5	S	7	830.8907
668.7084	6	S	6	743.8134
782.8111	7	N	5	656.7361
896.9137	8	N	4	542.6334
1010.0713	9	I	3	428.5308
1170.2356	10	C	2	315.3732
	11	K	1	155.2088

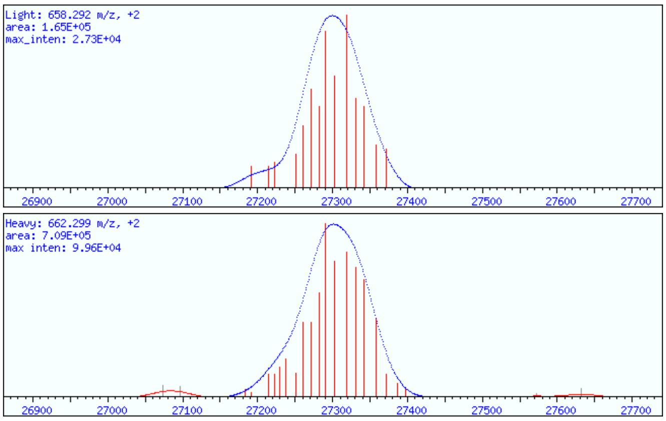

MS/MS and XICs for NCS6 peptide VSQLCELCHSR

sequence identified: K.VSQL$C_{160.03}$EL$C_{160.03}$HS$R_{166.11}$.K

m/z = 463.5518, z = 3

ratio(30 °C/37 °C) = Heavy/Light = 4.44

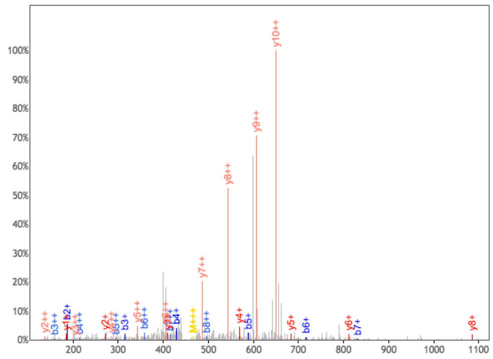

b+	b2+	#	Seq	#	y+	y2+
100.1383	50.5728	1	V	11		
187.2156	94.1115	2	S	10	1300.3973	650.7023
315.3449	158.1761	3	Q	9	1213.3200	607.1636
428.5025	214.7549	4	L	8	1085.1908	543.0990
588.6668	294.8370	5	C	7	972.0331	486.5202
717.7808	359.3940	6	E	6	811.8688	406.4380
830.9384	415.9729	7	L	5	682.7548	341.8811
991.1027	496.0550	8	C	4	569.5972	285.3022
1128.2420	564.6247	9	H	3	409.4329	205.2201
1215.3193	608.1633	10	S	2	272.2936	136.6504
		11	R	1	185.2163	93.1118

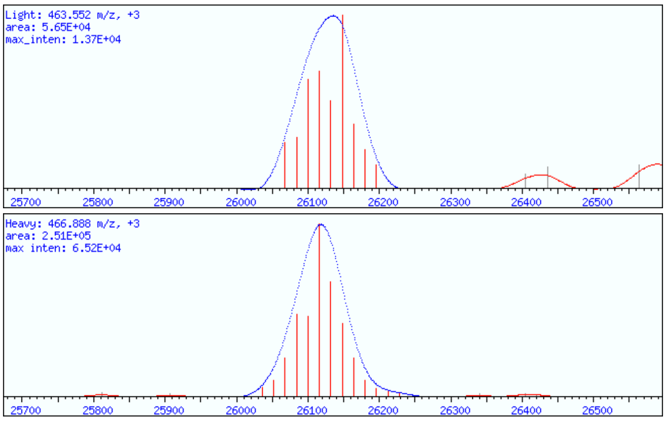

Supplementary Figure S6

A

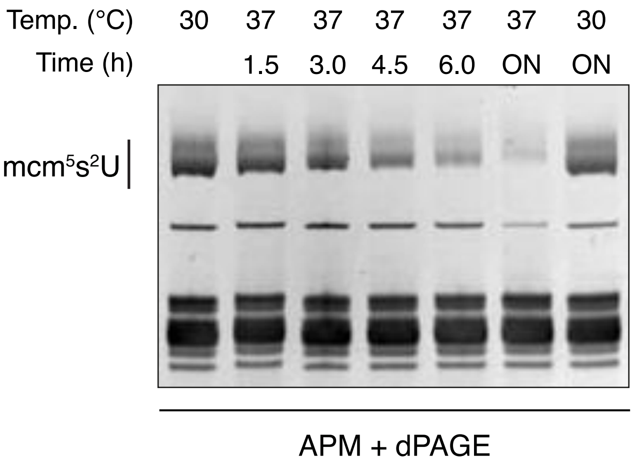

B

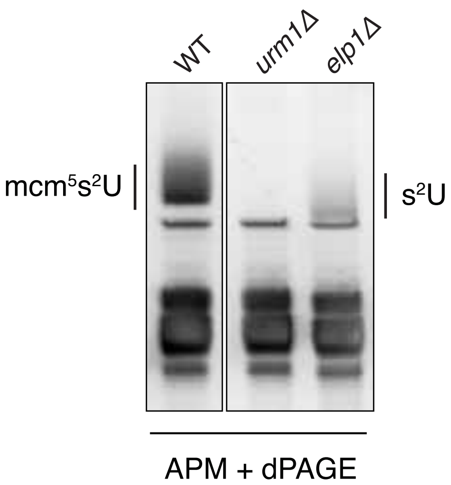

C

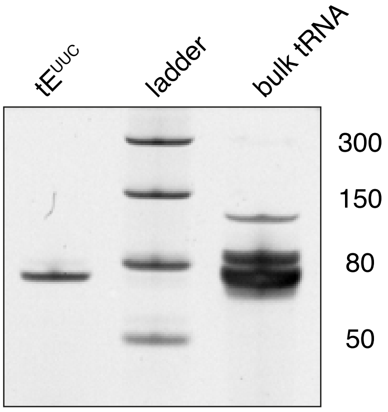

Supplementary figure S7

A

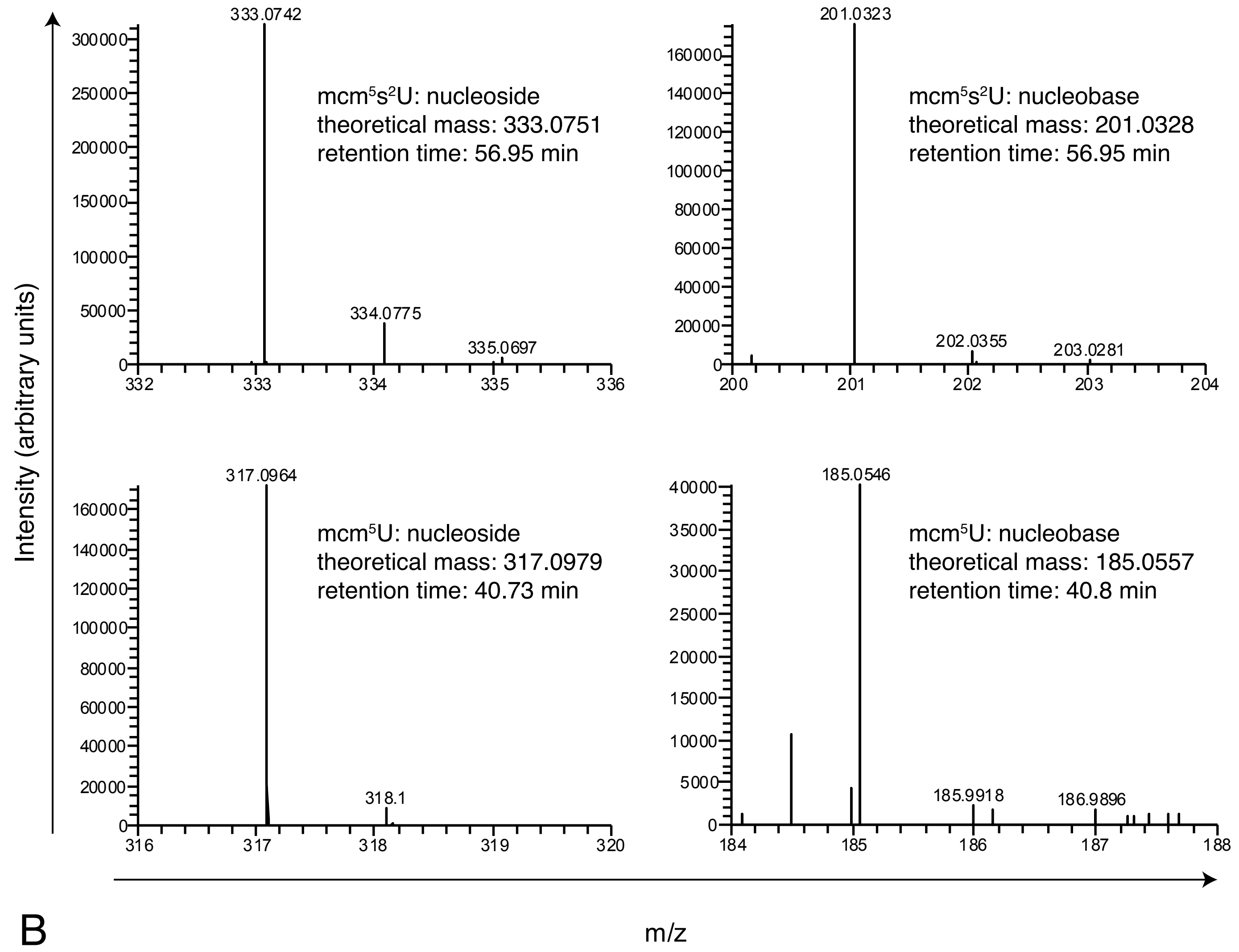

B

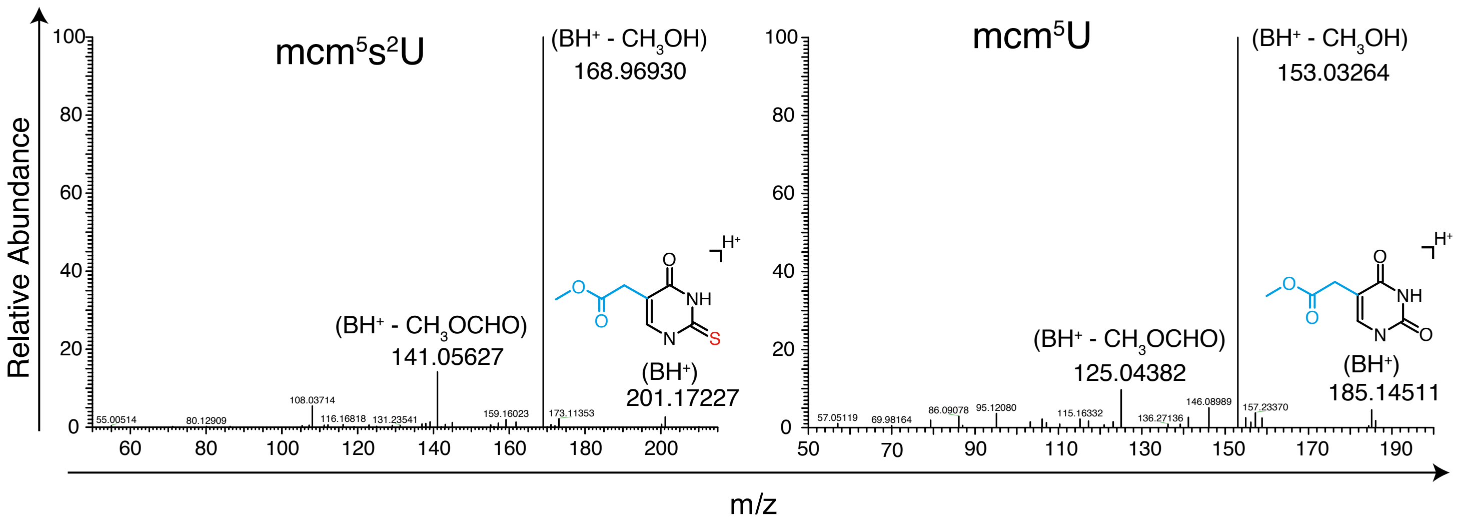

Supplementary figure S8

XICs for nucleoside and nucleobases from tQ^{UUG}

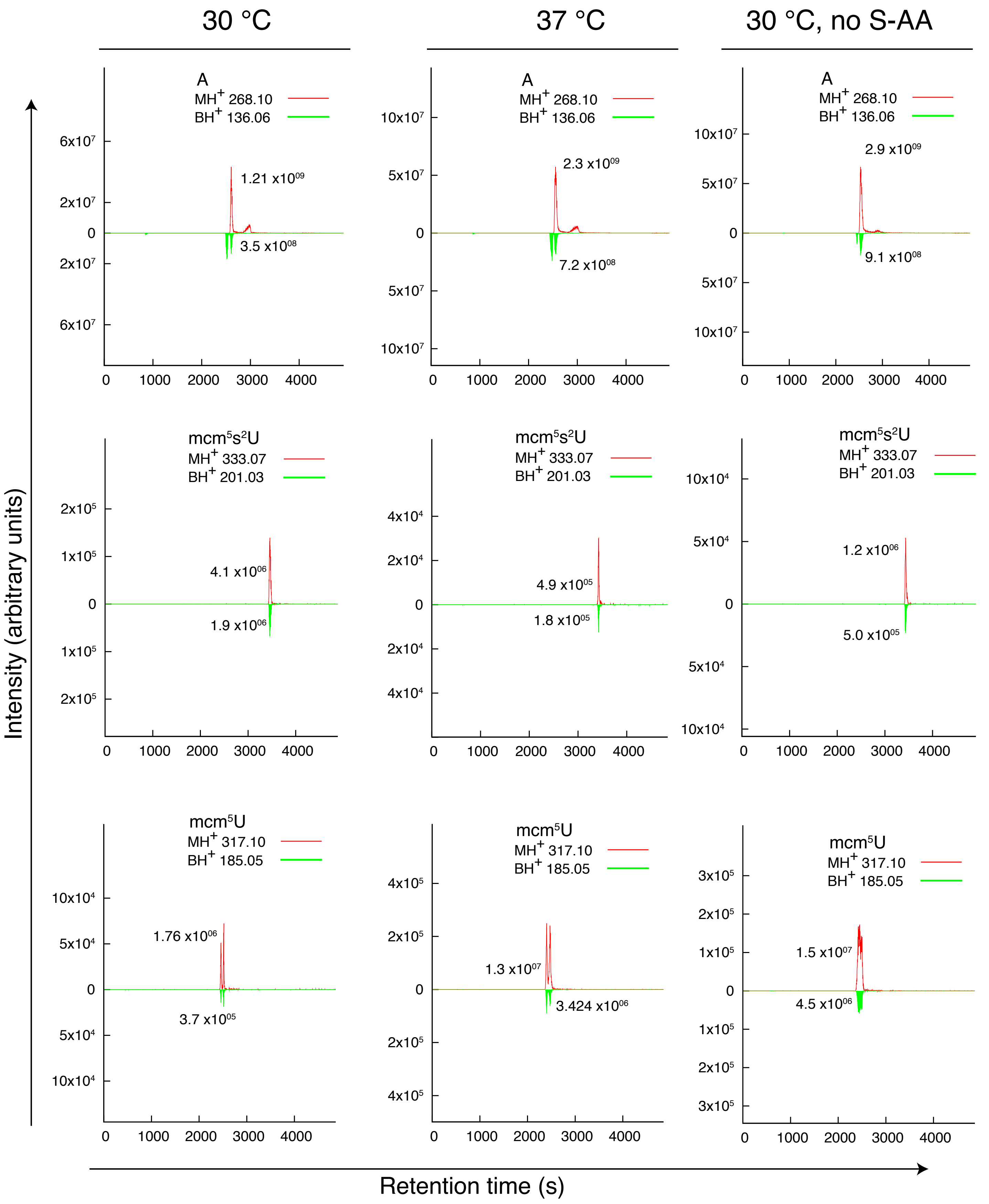

Supplementary figure S9

XICs for nucleoside and nucleobases from tE^{UUC}

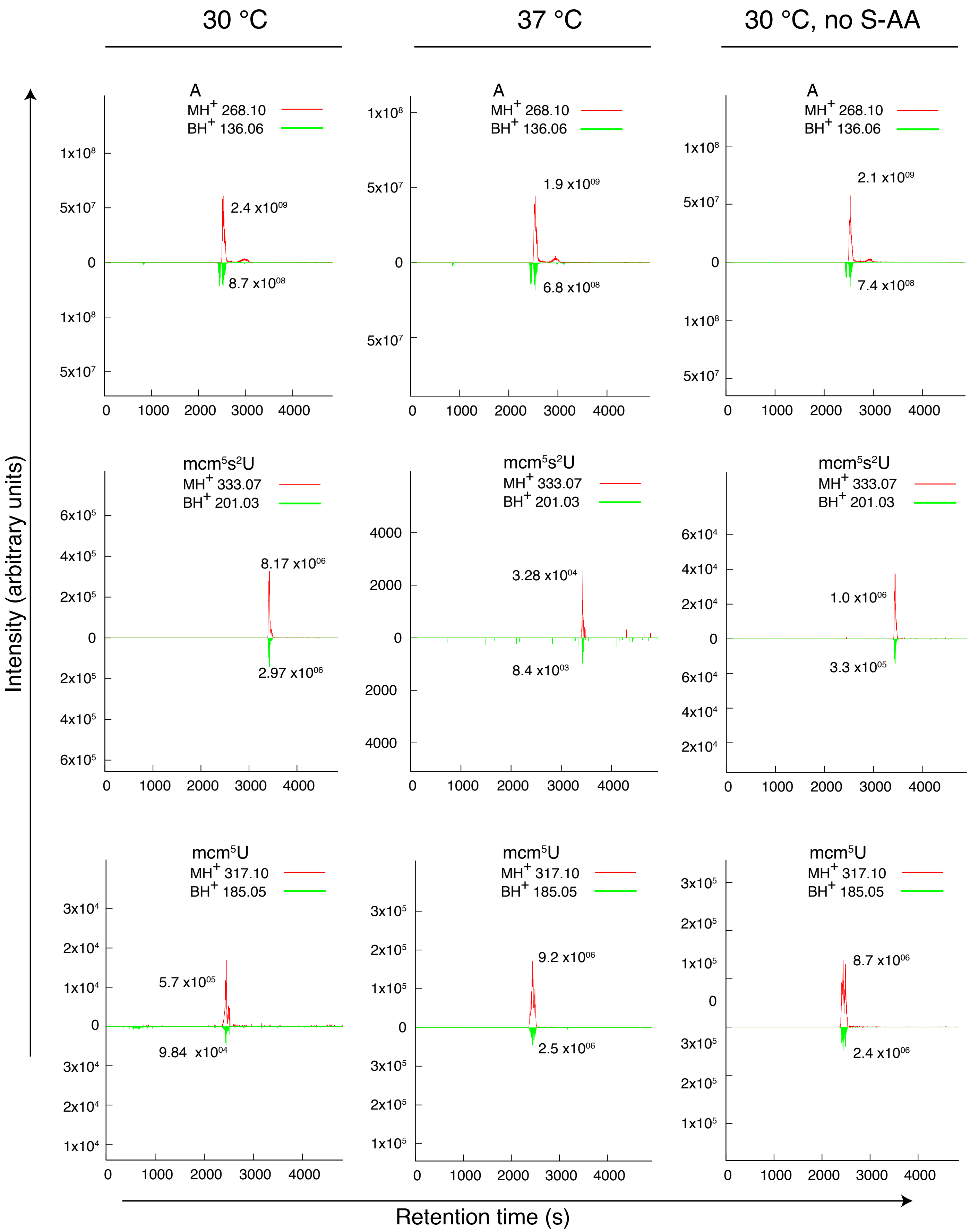

Supplementary figure S10

XICs for nucleoside and nucleobases from tR^{UCU}

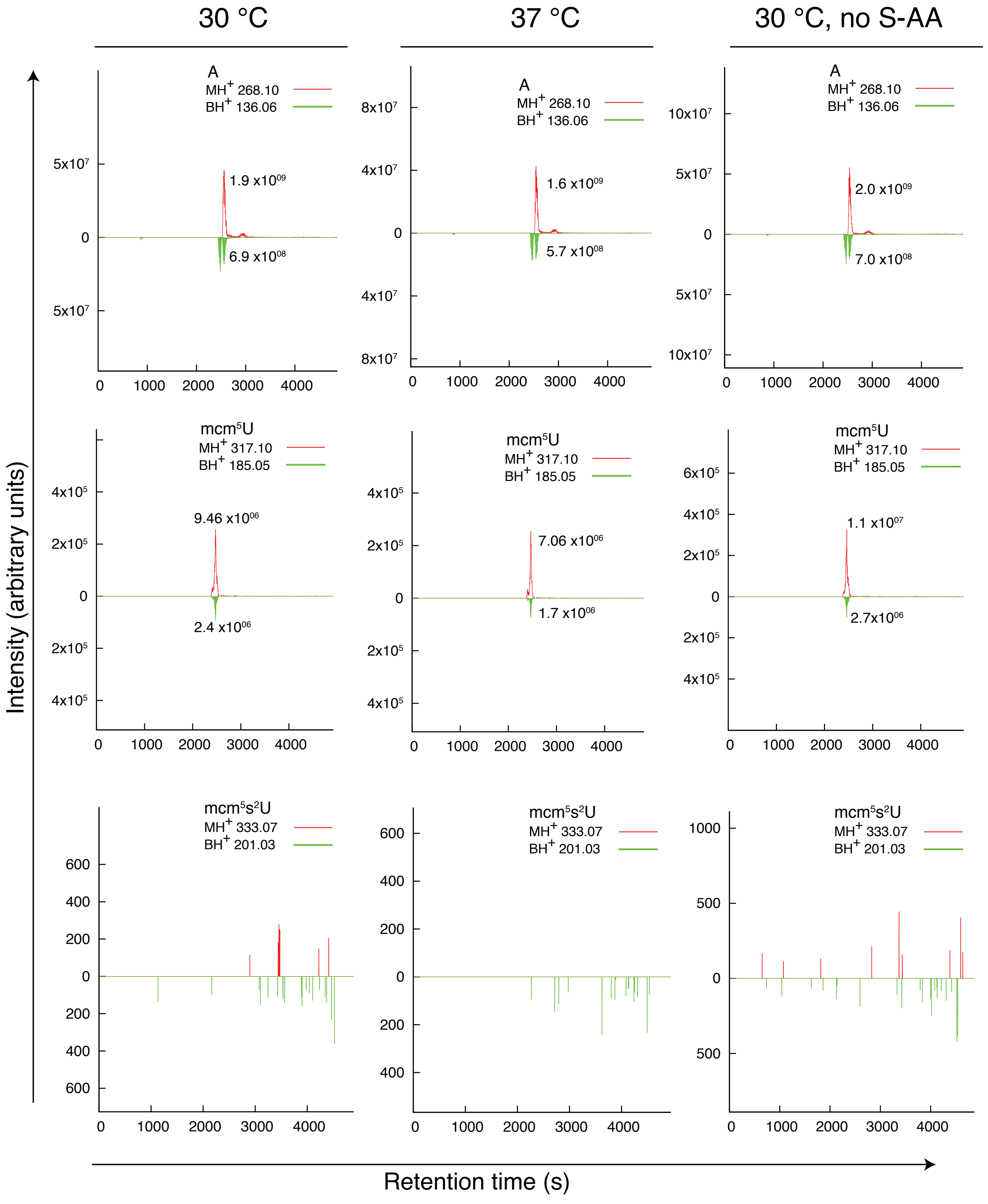

Supplementary Figure S11

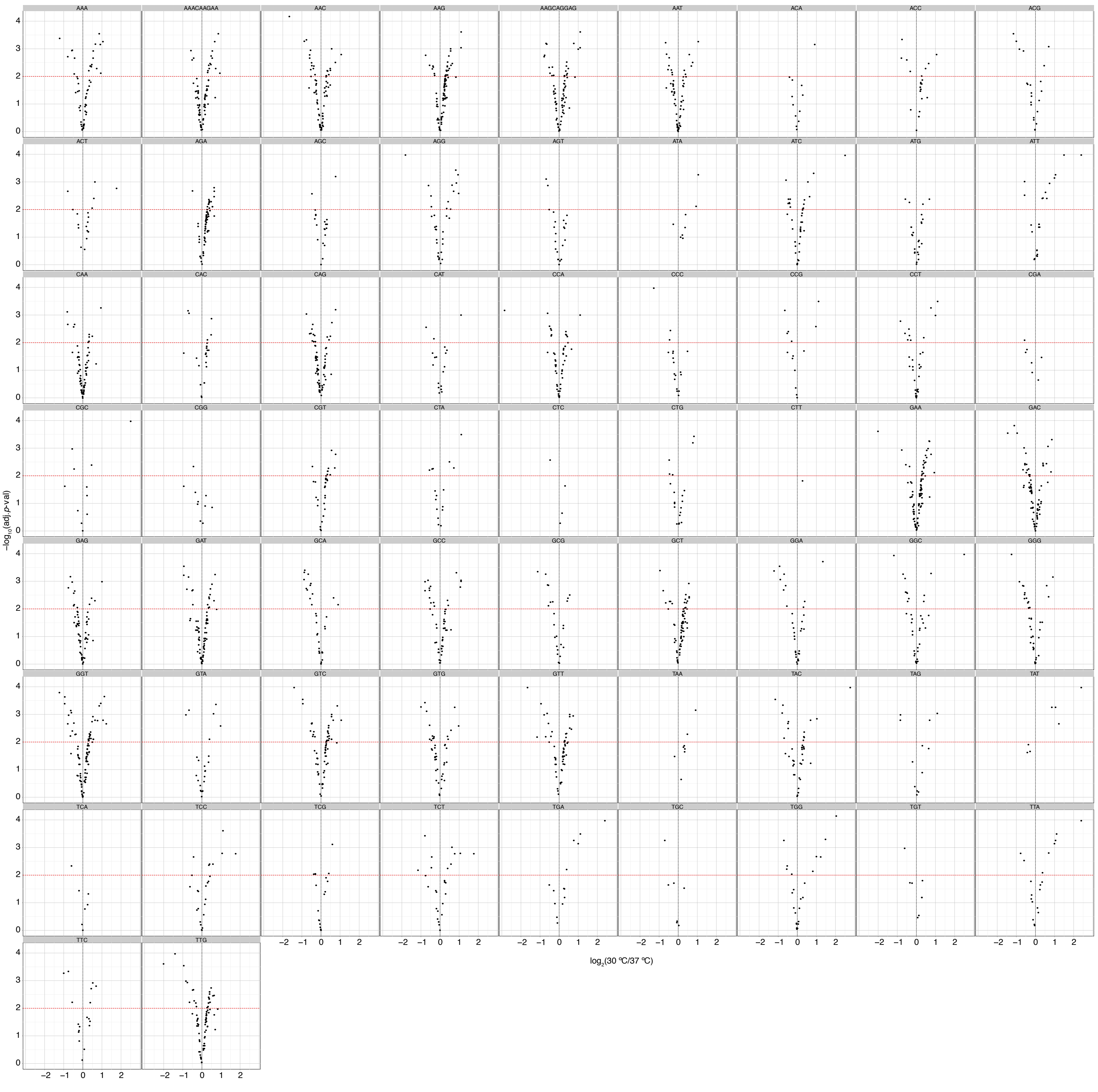

Supplementary Figure S12

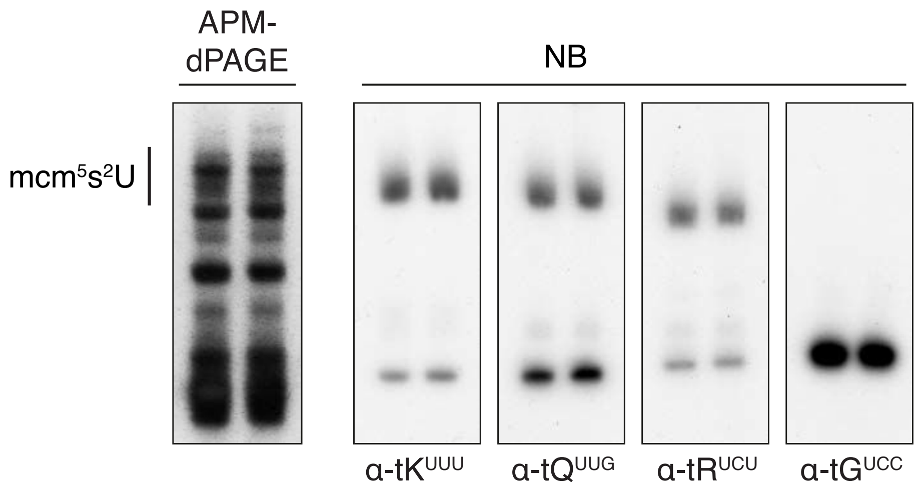

Supplementary Figure S13

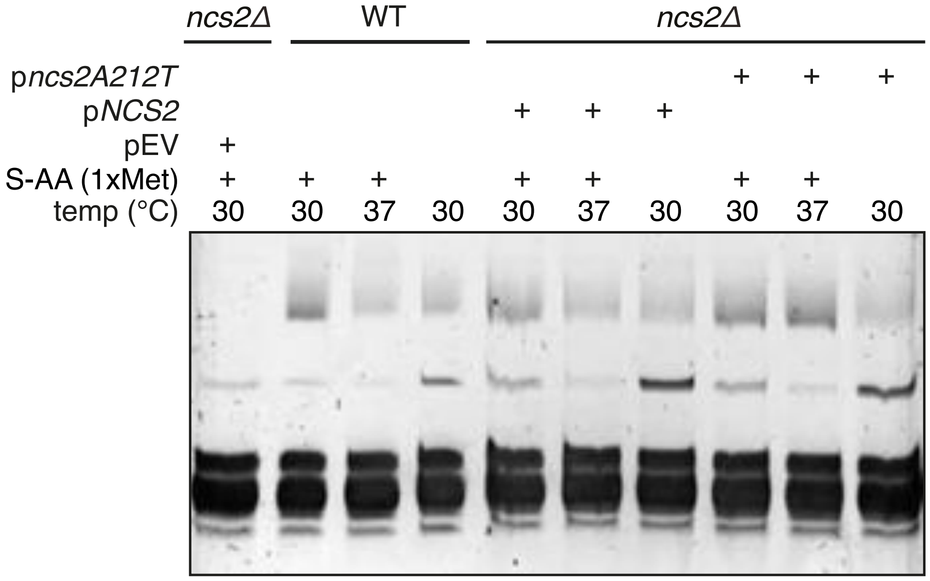

Supplementary figure S14

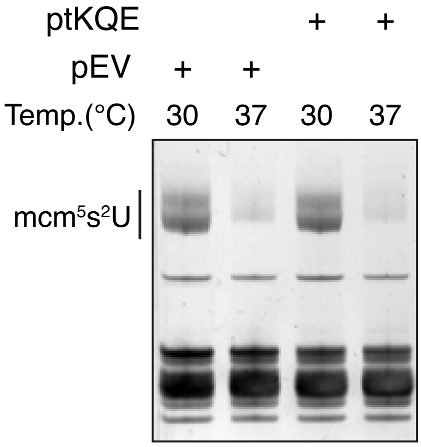

Supplementary Figure S15

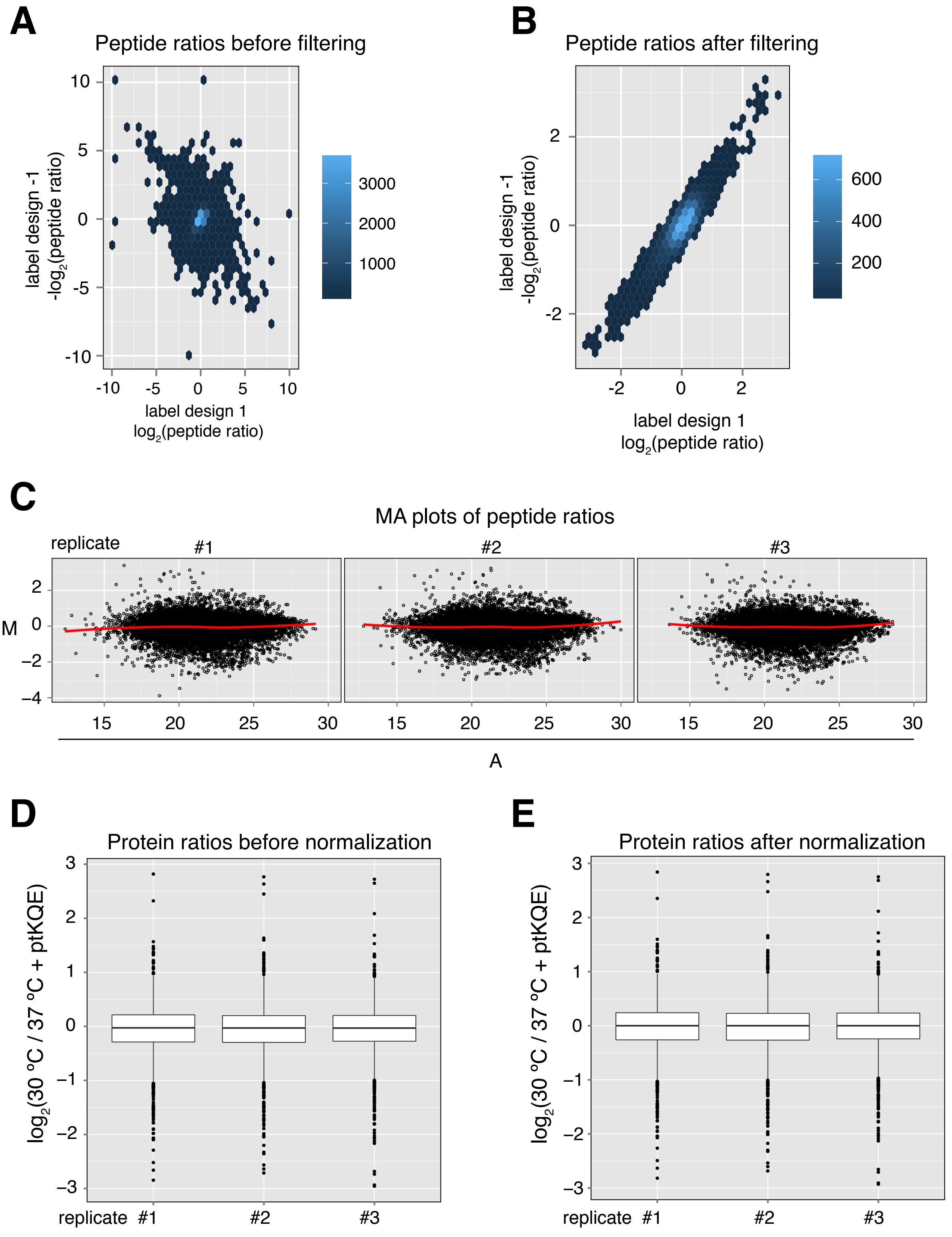

Supplement: SUPPLEMENTARY DATA [file supp_gkv322_nar-03542-f-2014-File007.pdf]
